# TAMEE: data management and analysis for tissue microarrays

**DOI:** 10.1186/1471-2105-8-81

**Published:** 2007-03-07

**Authors:** Gerhard G Thallinger, Kerstin Baumgartner, Martin Pirklbauer, Martina Uray, Elke Pauritsch, Gabor Mehes, Charles R Buck, Kurt Zatloukal, Zlatko Trajanoski

**Affiliations:** 1 Institute for Genomics and Bioinformatics, Graz University of Technology, Petersgasse 14, 8010 Graz, Austria; 2 Christian Doppler Laboratory for Genomics and Bioinformatics, Petersgasse 14, 8010 Graz, Austria; 3 Department of Mathematics C, Graz University of Technology, Steyrergasse 30, 8010 Graz, Austria; 4 ORIDIS Biomed GmbH, Stiftingtalstrasse 3-5, 8010 Graz, Austria; 5 Bindley Bioscience Center, Purdue University, 475 Stadium Mall Drive, West Lafayette, IN 47907, USA; 6 Institute of Pathology, Medical University of Graz, Auenbruggerplatz 25, 8036 Graz, Austria

## Abstract

**Background:**

With the introduction of tissue microarrays (TMAs) researchers can investigate gene and protein expression in tissues on a high-throughput scale. TMAs generate a wealth of data calling for extended, high level data management. Enhanced data analysis and systematic data management are required for traceability and reproducibility of experiments and provision of results in a timely and reliable fashion. Robust and scalable applications have to be utilized, which allow secure data access, manipulation and evaluation for researchers from different laboratories.

**Results:**

TAMEE (Tissue Array Management and Evaluation Environment) is a web-based database application for the management and analysis of data resulting from the production and application of TMAs. It facilitates storage of production and experimental parameters, of images generated throughout the TMA workflow, and of results from core evaluation. Database content consistency is achieved using structured classifications of parameters. This allows the extraction of high quality results for subsequent biologically-relevant data analyses. Tissue cores in the images of stained tissue sections are automatically located and extracted and can be evaluated using a set of predefined analysis algorithms. Additional evaluation algorithms can be easily integrated into the application via a plug-in interface. Downstream analysis of results is facilitated via a flexible query generator.

**Conclusion:**

We have developed an integrated system tailored to the specific needs of research projects using high density TMAs. It covers the complete workflow of TMA production, experimental use and subsequent analysis. The system is freely available for academic and non-profit institutions from .

## Background

Tissue microarray (TMA) technology is a high-throughput tool for simultaneous analysis of up to 1000 different tissue samples at the DNA, RNA or protein level in a single experiment. TMA technology evolved from different precursors [1-5] to a form described by Kononen *et al*. [6] and includes numerous varieties [7-10]. TMAs as proposed by Kononen consist of a paraffin block (array or recipient block) into which cylindrical tissue cores extracted from different formalin-fixed paraffin-embedded (FFPE) tissue samples (donor blocks) are inserted in an array. From a single array block up to 200 sections can be made and analyzed by immunohistochemistry (IHC), *in situ *hybridization (ISH), or immunofluorescence (IF).

Compared to conventional pathology analyses where single tissue specimens are investigated individually, TMAs provide results from large numbers of different tissue samples in a single experiment. The major advantage is that the experimental conditions for all samples are equal and the amounts of consumables required are markedly reduced. TMAs also preserve precious raw material (archived tissue samples) since a single donor block can provide cores for multiple arrays [11,12].

The TMA workflow comprises the following steps: (i) design of the TMA and selection of tissue samples, (ii) production of the array block, (iii) sectioning of the block and quality control of the sections, (iv) molecular analysis of TMA sections, (v) image acquisition, (vi) evaluation of core images, and (vii) statistical analysis of the results. These steps produce a significant amount of data that must be stored in a centralized repository to give all researchers involved in a TMA-based study access to the relevant information. Special emphasis has to be directed to the standardization of the entered data through the use of existing ontologies and classifications to enable meaningful down-stream analysis. Sample scoring has now become the major bottleneck since instead of a small number of stained whole biopsy sections, hundreds of cores must be evaluated in a reproducible and timely manner.

The power of the TMA technology has led to a rapid increase in the number of labs adopting the technology and an explosion of generated data. Managing and extracting valuable information from such data requires new and efficient data management platforms and computational approaches. Numerous efforts have been undertaken to develop software tools to handle different aspects of the TMA workflow. However, to the best of our knowledge, there is currently no academic data management platform supporting the complete TMA pipeline. Among existing applications spreadsheet based systems [13-16] have been suggested as well as applications concentrating on web based manual core evaluation [17-20] and applications with a wider range of workflow components [21-27]. The applications of Chen *et al*. [28,29] and Rabinovich *et al*. [30] focus mainly on the image acquisition step. Only Vrolijk *et al*. [15] and Demichelis *et al*. [25] provide systems that contain a component for automatic core localization and extraction of core images (see Table S1 in Additional file 1 for comparison of features of currently published systems).

One of the most important steps in the TMA workflow – the evaluation of core images – has been addressed in a single database application [25]. The results of the evaluation are the basis for the downstream analysis, where biological markers are correlated with outcome and prognosis or used to determine therapeutic measures. Manual evaluation has been shown to be prone to significant inter- and intra observer variability, especially for borderline cases and with less experienced clinicians [31]. Automated image analysis promises to reduce evaluation variability [31] and to reveal novel findings, not detected by traditional pathologist-based scoring [32]. Different (semi)-automated evaluation algorithms have been described for IHC [15,29,30,33-35], IF [30,32,36,37] and for ISH experiments [38]. Only the algorithm from Dell'Anna *et al*. [35] is integrated into a web-based TMA data management application [25].

Given the main shortcomings in the area of quality control, image acquisition and automated core analysis sparked us to develop TAMEE, a web-based database application using state of the art software technology, ensuring extensibility, scalability, maintainability and portability. It supports the complete TMA workflow, comprising the following features:

• Management of data related to tissues and donor blocks

• Storage of TMA design and array block data

• Tracking of array block sectioning and section quality control

• Automated gridding of the TMA slide images and extraction of core images

• Automated evaluation of core images and flexible storage of evaluation results

• Plug-in framework for the integration of additional evaluation algorithms

• Support for standardized and consistent database content with controlled vocabularies

• Compatibility with data interchange standards

• Operating system and browser independence

## Implementation

### System architecture

The TMA data management application was implemented using Java, an object-oriented and platform independent programming language [39]. The application is three tiered, consisting of the database tier, the application server tier and the client tier. The open source database MySQL [40] is used as the data-base backend and the business logic is implemented using Enterprise Java Beans (EJBs) [41]. The runtime environment for the EJBs is provided by JBoss, a J2EE compatible application server [42]. The presentation layer is implemented using the Model-View-Controller (MVC) framework Struts [43]. Features requiring complex, immediate response on user input are implemented as Java Applets [44].

Integration of algorithms evaluating core images is facilitated by a plug-in framework in a flexible and extendable way. The framework consists of the following components: (i) an abstract algorithm bean, which defines the methods to be implemented and provides convenience functions which can be utilized by the derived algorithms, (ii) a flexible database model to manage the available algorithms and to store the image evaluation results (see below), and (iii) a web interface to hot-deploy newly created algorithms and to manage existing ones. A description of the framework and Java source code for a sample algorithm is provided as Additional file 2. It can be used as the basis for the development of additional algorithms. Commercial algorithms can be integrated by implementing a "wrapper" algorithm, provided a command line version of the commercial algorithm exists.

The complete application has been designed using UML [45]. The UML representation improves maintainability as the application architecture is immediately visible and the components can be easily identified. The UML model is the basis for code generation with the AndroMDA framework [46], which relieves the developer from tedious and repetitive coding task and allows him to concentrate on the business logic and presentation part of the implementation. The UML model is available as Additional file 3.

### Database schema

The database schema supports the complete TMA workflow (see Additional file 4). TMA design and construction is covered by the "Tissues", "Donor" and "Arrayblock" tables. The tissue tables are accompanied by the tables storing the ICD-O diagnosis coding and the TNM staging definitions in the data dictionary. Additionally de-identified sample identification is stored, which provides a link to both sensitive and insensitive patient data in the clinical database. The array layout including the array attributes like core diameter and spacing is associated with a constructed array block. The "Section" table stores data related to a cut section like related array block, thickness, section index within array block, and type of microtome used. One or more section images (stained or unstained) can be related to a section via the "SectionImage" table. For stained sections, the staining protocol, the antibody used and the related antigen are recorded. Each core stored in the "Punch" table is associated to a specific section, the corresponding array block and the related donor. A list of available evaluation algorithms is located in the "Algorithm" table; the evaluation results of core images in the "PunchImage" table are stored in the "Result" table.

A hybrid database schema has been designed consisting of a conventional relational part for entities with a static attribute set and a part implemented as an entity-attribute-value (EAV) schema [47,48]. In the former, each parameter is represented by a separate column in a database table. The latter is used to support the highly dynamic attribute set resulting from the manual and automatic evaluation of core images. The entity is a certain core image, which has been evaluated, the attribute is the algorithm used for the evaluation of the core image and the value is the result of the evaluation. A simplified EAV approach is used, where all values are stored as strings regardless of their actual data type. Using the EAV approach to model the evaluation data, results from new, currently not foreseen scoring algorithms can be accommodated in a generic way, without the necessity to update the database schema.

Controlled vocabularies are stored on the one hand in the ICD-O related tables ("Topography", "Morphology", "Behaviour" and "Grade") and on the other hand in the "Datadictionary" table.

All images are stored as binary large objects (BLOBs) in the database using two formats, namely resolution reduced, compressed JPEG for quick viewing and high resolution, lossless compressed TIFF for scoring and display of image details.

### Image acquisition and extraction of core images

Images of the HE stained donor block sections, which are used to determine the positions where the cores are extracted and the TMA sections used for quality control are scanned with an Epson Perfection 1660 Photo flatbed scanner (Epson, Meerbusch, Germany) with 16 μm per pixel. IF-stained sections were digitized with a GMS-418 laser scanner (Affymetrix, Santa Clara, CA, USA) at 10 μm per pixel.

Extraction of core images is a three step process: (i) marking of corner cores position (ii) localization of cores in the image, (iii) determination of core extents, and (iv) saving of core images in the database. Prior the determination of the core positions, color images are converted to grayscale, taking the different luminance contributions of the colors into account. Corner core positions are marked manually to be able to determine the overall array orientation. After cropping, mirroring, and rotation of the image based on the marked corner positions, the centers of the cores are determined using the radon transform [49]. Based on the array layout (the number of rows and columns) the local minima in the radon transformed data are used as the starting points for horizontal and vertical lines. The crossings of the lines are regarded as approximate centers of the cores. A binary image is created based on the gray-scale histogram and noise is removed by morphological image operations. The previously determined core centers are used as the seed for the subsequent border tracing algorithm [50] to determine the exact extent of the core in the image. Rectangular sub-images which cover the complete core are extracted from the section image and all pixels outside the cores extend are set to 0 before the image is stored in the database.

### Section quality control

After a section has been cut and transferred to the carrier slide, it is scanned with a flatbed scanner to keep track of the section quality. These quality control images are gridded and the images of the cores extracted. For each core, the nominal core area (calculated based on the core diameter from the array layout) is compared to the actual area. If the actual area is below (indicating loss of tissue) or above (indicating overlapping or aggregation of cores) a certain percentage of the nominal one, the core is flagged as bad. Additionally, cores whose shape significantly deviates from circularity are marked.

### Analysis of multiple IF stained cores

Double stained sections using antibodies labeled with different fluorophores (e.g. Cy3 and Cy5) are scanned with a laser scanner, which creates two grayscale images, one for each fluorophore [37]. A merged image is gridded and the core images are extracted from both original images as described above. The individual core images are evaluated with respect to the following parameters: median core intensity, integral of core intensity and percentage of core area stained. Additionally, the ratio of the median intensities of the two fluorophores, the percentage to which the stained areas of the two fluorophores overlap, and the median intensity of one fluorophore in the area which is stained by the other are calculated. The evaluation results are stored in the database, where they are available for further analysis.

## Results and discussion

TAMEE is a system which covers all steps in the TMA workflow, starting with the design of a TMA and spanning to the automated analysis of core images. It accommodates the requirements of different tissue types and of current and future image evaluation algorithms. The web-based application uses J2EE application server technology providing industry grade stability, scalability, and extensibility.

### Tissue microarray design

**Figure 1:**
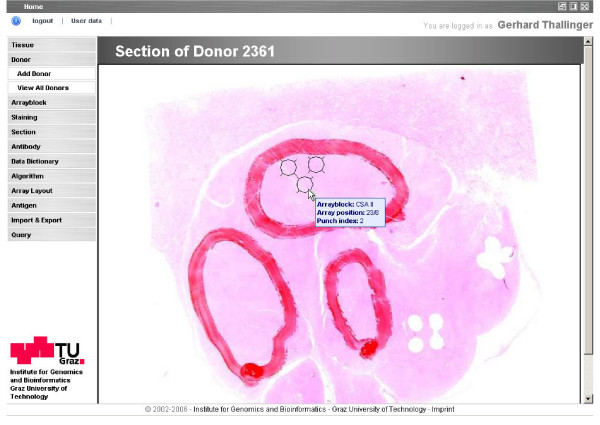
**HE stained section of a donor block**. The section is used to identify areas of interest in the donor block for extraction of cores to place in the array block (identified by a pathologist with red or black ink). The positions where cores have been extracted are marked with black circles. Moving the mouse over a core position displays detailed information about the related array block, the position of the core on the array block and the index of the core on the donor block.

Prior to the production of a TMA, tissues appropriate for a given TMA design are selected by querying the clinical database which contains information regarding cases and associated donor blocks. Basic tissue information (like diagnosis and unique sample identification) is exported from the clinical database in tabular form. This table is amended with the bar-code identifier of the donor block and specific comments about core selection and location. The sample data related to an array is uploaded into the database and joined after array production with the layout information. Alternatively, tissue and donor block information can be entered manually if needed. Images of donor block sections stained with Hematoxylin and Eosin (HE), used to identify a representative core position within the specimen, are also stored in the database for quality control and tracking purposes (Figure 1).

### Production and quality control

**Figure 2:**
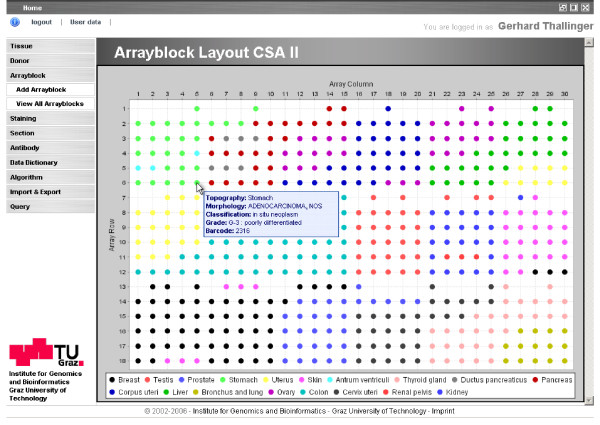
**Layout of a TMA with 487 cores**. The individual cores of the array are colored according to the topographic origin of the tissue sample. Moving the mouse over a core displays detailed information about the core: topography, morphology, classification, grade (according to ICD-O) and the barcode of the donor block.

Array blocks are produced with a custom-built robotic TMA Arrayer (Oridis-Biomed GmbH, Graz, Austria), based on the selected position of a core on the donor block and the target location for the core in the array block. Each array block is also identified by a unique barcode. During array production, the robot control software produces an array description file in tabular form, including the donor block barcode, core location on the donor block, and row and column of the core in the TMA. This file is uploaded into the database and associated to the corresponding donor blocks and to one of several existing array layouts possible with the arrayer. Currently four layouts encompassing 240 to 487 cores are available (Figure 2). Additional array layouts can be defined via the web interface.

**Figure 3:**
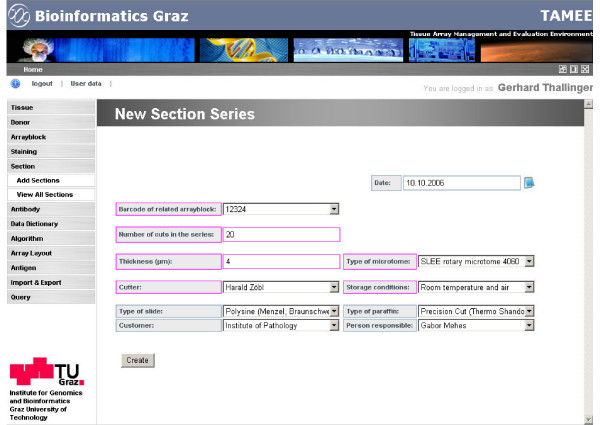
**TAMEE sample input screen**. Fields where input is required are marked magenta, optional fields are colored blue. Drop-down list boxes for the selection of appropriate attribute values are used extensively to ensure standardized and consistent database content.

Sections created from the TMA block are recorded in the database together with related information like section thickness, type of microtome used, and corresponding depth in the TMA block. Generally, sections are created in batches; this is accounted for in the section entry screen, where section parameters of a batch including the number of sections are defined (Figure 3). To track the quality of the cut sections, a flatbed scanner is used to generate a low resolution image of the unstained slide. These images are stored in the database and quality scores that include, for example, core completeness and core shape are assigned via an evaluation algorithm.

### Molecular section analysis

TMA sections are useful for a range of molecular analyses that can also be performed on regular tissue sections, such as IHC, ISH, or IF. For every analysis the subject of the analysis (antigen, DNA or RNA sequence) and the experimental conditions are recorded. Type and concentration of antibodies utilized in an IHC or IF analysis are added as an experimental parameter. To ensure reproducibility, the experimental protocol is associated in TAMEE as a PDF file for reference during data evaluation. Digital images of the stained section, obtained either via photomicroscopy or from a laser scanner, are related to the analysis parameters and stored for later core image extraction and analysis. In general one grayscale or color image is available from an experiment, in the case of IF experiments with multiple antigens investigated, two grayscale images are created for joint evaluation [37].

### Extraction and evaluation of core images

**Figure 4:**
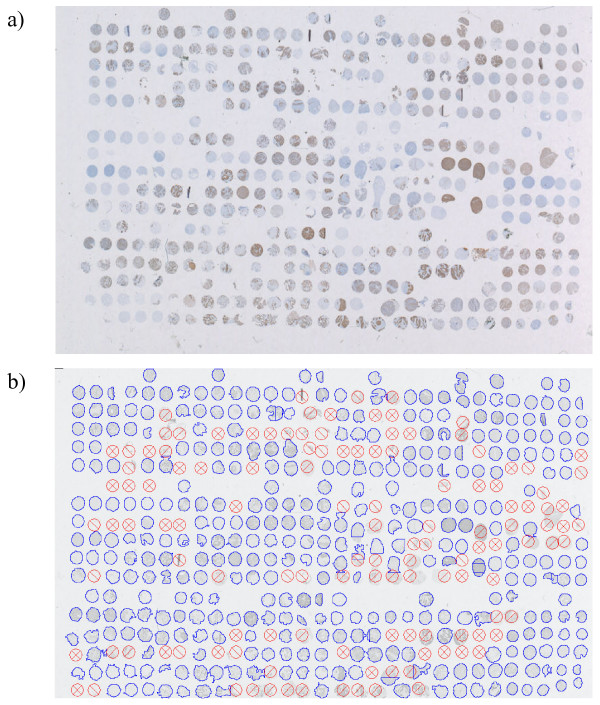
**Stained and gridded TMA section**. (a) TMA section with about 480 tissue cores from different cancer tissue samples. Cytokeratin has been detected by IHC (brown areas) using a cytokeratin antibody (mouse anti human; DAKO; M3515) and the EnVision detection Kit (DAKO, K4065). Tissue cores are made visible with a Hematoxilin counterstain (light blue). (b) The same section after automatic core localization and shape determination. The outline of a core is displayed in blue. Positions with missing cores (⊗), cores which are too large (more than 160% of the expected area, ) and cores which are too small (less than 40% of the expected area, ) are shown in red. Array positions without a symbol or outline have been deliberately left empty during TMA production. Gridding ambiguities in heavily distorted areas of the array are corrected manually.

Images of a complete TMA section are uploaded into the database. During core image extraction the individual cores are automatically located (Figure 4) and a square image area with the core in its center is stored for later analysis. Extraction from both HE or IHC stained images (with a white background) and IF images (with a black background) is supported. Pixels not belonging to the core (either background or artifact) are set to zero to provide a standardized basis for core analysis. Gridding and extraction of cores from an image of a TMA with 487 cores is accomplished within 35 seconds on an Intel Pentium M system at 1.6 GHz and 1 GB of RAM.

Cores originating from the quality control section images are evaluated for area and shape (roundness). Cores that fall within certain area and roundness ranges are considered acceptable for evaluation in later experimental analysis. If a section shows too many cores outside the acceptable range, the section is abandoned.

IF experiments allow the concurrent investigation of multiple antigens using different fluorophores attached to the antibodies to determine, for example, colocalization of a tumor marker with a cell type specific marker. For each fluorophore, a grayscale image is created, from which the area stained by the antibody is determined. Additionally, the area of overlap of staining with the different antibodies is calculated. This allows normalization of the results and semi-quantitative analysis of the cores [37].

For each run of an analysis algorithm, evaluation results are written to the database together with the evaluation results, date and time of the run and a reference to the user who invoked the analysis. There is no restriction on the type of evaluation results, categorical data as well as continuous data is supported. The analysis algorithms are embedded in a plug-in framework, which allows the integration and use of additional algorithms without requiring adaptations in other parts of the application (see Additional file 2 for a description of the framework and a sample algorithm). These additional analysis algorithms can either be newly developed academic ones or existing commercial ones like AQUA [51] or SpotBrowser [52].

Automated core analysis provides several advantages over manual scoring which is affected by intra- and inter-observer variability and allows assignment of categorical scores only. Automated image analysis improves the reproducibility of the evaluation results [31,53] and provides a solid basis for subsequent analysis of the prognostic value of a certain marker [54]. Furthermore, scoring is carried out on a continuous scale, which allows the analysis to be performed without stratification. This increases the statistical power of the analysis and avoids the introduction of a bias [55,56].

### Controlled vocabularies

User input for the same entity can be very heterogeneous, depending on educational background, personal preferences and a host of other variables. Non-standardized data is almost impossible to query and therefore diminishes the value of the database. Hence TAMEE uses – wherever possible and reasonable – existing public classifications and self-defined data dictionaries to ensure standardized and consistent data entry. Disease diagnoses are encoded using ICD-O (International Classification of Diseases for Oncology) [57] and the TNM classification scheme [58] is used for the tumor status. Custom data dictionaries have been created for TMA parameters including tissue source, type of paraffin utilized, microtome model used during sectioning, and section storage conditions. The data dictionaries are user extendable, which allows immediate modification of parameter lists without adaptation of the application itself.

### Data entry and query

**Figure 5:**
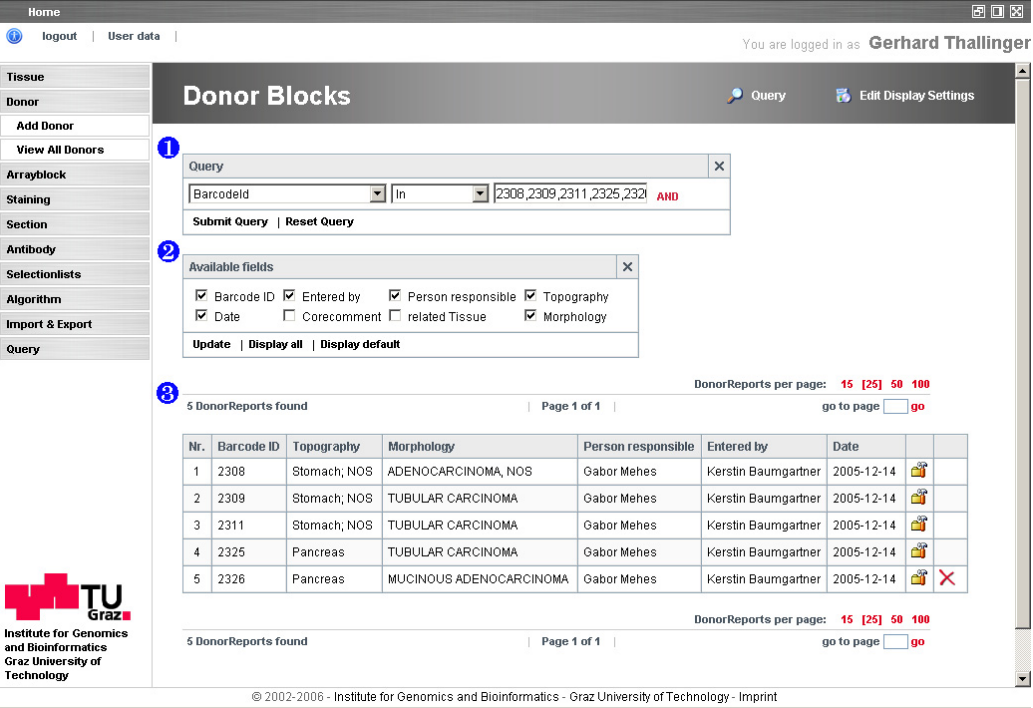
**Sample TAMEE list view**. A selection of donor blocks based on their barcodes is shown. The list view consists of 1) the query parameters, 2) the customizable display settings and 3) the list itself. Areas 1) and 2) are hidden by default and are made visible by clicking at the respective icons in the header.

A consistent and clear application interface is provided for users with primarily a medical or biological background. Screens can be divided into two major groups: (i) entry screens for primary data entry, and (ii) list views for the compact display of available entities. Entry screens consist of mandatory fields – colored magenta- and optional fields in blue (Figure 3). Drop down selection lists are used extensively to ensure standardized data entry. Free text can be entered in fields not used in subsequent analysis queries. Data entered is checked for validity and in the case of an erroneous entry the user is informed appropriately. List view screens present the entities in tabular form and allow paging, querying and the selection of which entity attributes are displayed (Figure 5). Queries can be performed on any combination of the available attributes using a standard set of query operators. After the query is submitted, the displayed list is narrowed to the matching entries of the specified query.

### Data access control

Although TAMEE manages de-identified patient data only, access to and modification of the information has to be tightly controlled. Therefore, access to the web application is secured by an authentication and authorization system as described previously [59]. It manages user account information, assignment of users to certain application roles and application resources together with their associated access control lists. By default, TAMEE utilizes three application roles, namely "Administrator", "User", and "Guest". Guest users are granted read only access to all entities. The more privileged User role allows performance of all operations related to the routine TMA workflow. Administrators are enabled to perform management tasks like altering and extending the data dictionary and the definition of array block layouts. Additionally, the right to delete data in the database is granted to administrative users only. The access rules for these groups can be changed according to different requirements and additional groups with special entity access patterns can be defined using the authentication and authorization system.

### Support for data exchange specifications

To allow sharing of experimental data in a reproducible and complete form, data interchange standards are becoming increasingly important [60,61]. To this end, the Association for Pathology Informatics proposed the "Tissue Microarray Data Exchange Specification" (TMA-DES) [62-64]. TAMEE can export TMA analysis data in the format proposed in TMA-DES and allows the dissemination of supplemental experimental data related to a publication and the submission of parameters and results to public data repositories in a standardized format. Recently, the "MISFISHIE" standard [65] covering IHC and ISH experiments has been described and TMA-OM [27], an object model for TMA data accompanied by a corresponding XML representation has been suggested. As these data representations gain acceptance, support for them can be added to TAMEE as well.

## Conclusion

We have developed TAMEE, a portable, web-based database application using state of the art application server and database technology tailored to the specific needs of research projects using high density tissue microarrays. The system covers the complete TMA workflow and manages significant amounts of data accumulating during the production and application of TMAs. It allows simultaneous access and update of data as required in multicenter studies. TAMEE facilitates the traceability of all steps during the production and the molecular analyses of TMA sections by collecting the relevant laboratory and quality parameters. Additionally, TMA data can be exported in the standardized TMA-DES format for sharing and dissemination of TMA design, experimental conditions, and evaluation results. Key features are quality control in TMA production and molecular analysis, standardized data entry using controlled vocabularies, and the plug-in framework for the integration of automated core image evaluation algorithms.

## Availability and requirements

• Project name: TAMEE

• Project home page: 

• Operating system: Solaris, Linux, Windows, Mac OS X

• Programming language: Java, HTML

• Other requirements: Java JDK 1.5.x, MySQL 4.0.x with InnoDB engine, a server with at least 1 GBytes of main memory available to the application

• License: None required

• Any restrictions to use by non-academics: None

Installation of the application is straight-forward and should be manageable within a few hours provided the necessary MySQL access rights are granted. Step-by-step instructions are provided at the projects Web site together with the files and scripts necessary for installation. The reference installation of TAMEE is running on a Sun V20z Opteron server (Sun Microsystems Ges.m.b.H, Vienna, Austria) with 8 GB of memory under CentOS Linux [66] using MySQL [40] as database management system. Attached is a Storage Area Network (EVA 5000, Hewlett-Packard Ges.m.b.H., Vienna, Austria) with 5.5 TBytes.

The production instance of TAMEE is hosted on a HP Proliant ML350G4P (Hewlett-Packard Ges.m.b.H., Vienna, Austria) under Windows 2003 Server with 2 GB of main memory and an 800 GB RAID-5 storage.

## List of abbreviations

BLOB Binary Large Object

EAV Entity Attribute Value

FFPE Formalin fixed, paraffin embedded

ICD-O International Classification of Diseases for Oncology

HE Hematoxilin and Eosin

IF ImmunoFluorescence

IHC ImmunoHistoChemistry

ISH *In Situ *Hybridization

J2EE Java 2 Enterprise Edition

JPEG Joint Photography Expert Group

TMA-DES Tissue MicroArray Data Exchange Specification

TIFF Tagged Image File Format

TNM Tumor, Nodes, Metastasis (tumor classification system of the Union against Cancer)

## Authors' contributions

GGT designed the application, implemented the section quality control and drafted the manuscript, MP and KB implemented the business – as well as the presentation logic. MU developed the automated gridding of the section images including the extraction of the core images and EP implemented the two color analysis algorithm. GM designed the cancer survey array and defined quality control procedures. CB and KZ participated in the compilation of the user requirements and contributed to the conception and design of the system. ZT was responsible for the overall project coordination. All authors contributed in reading and revising the manuscript and gave final approval of the version to be published.

## Supplementary Material

Additional File 1 **Table with features of published TMA applications**. PDF file containing a table showing the features of TMA applications described in literature. Click here for file

Additional File 2 **Sample core evaluation algorithm**. Zip file containing Java source code of a sample core image evaluation algorithm and a description of the plug-in framework as the basis for the development of additional algorithms. Click here for file

Additional File 3 **TAMEE UML model**. Zip file containing the TAMEE UML model comprising the entity and the service diagrams. Click here for file

Additional File 4 **TAMEE entity-relation diagram**. Bitmap file containing the TAMEE entity relation diagram. Click here for file
