# Supplementary material for: TAMEE: data management and analysis for tissue microarrays
Source: BMC Bioinformatics. 2007 Mar 7;8:81. doi: 10.1186/1471-2105-8-81 (PMC1838435; doi:10.1186/1471-2105-8-81)
Supplement: Additional File 1 — Table with features of published TMA applications. PDF file containing a table showing the features of TMA applications described in literature. [file 1471-2105-8-81-S1.pdf]

Table S1: Feature overview of published TMA management applications sorted by publication date

Supported features / Application name [Reference]	Clinical data	Tissue parameters	Multi species support	TMA layout data	Storage of donor images	Section tracking	Section quality control	Molecular analysis data	Storage of section and / or core images	Automated gridding	Manual Scoring	Automatic Scoring	Supported tissue types	Export as TMA-DES [1,2]	Export as MISFISHIE [3]	# of user roles	Relational Database	Controlled vocabularies	Browser based access	Platform independent	Publicly available
Bova *et al.* [4]	-	-	-	-	-	-	-	✓	-	-	✓	-	1	-	-	1	✓	✓	✓	-	-
Manley *et al.* [5]	✓	✓	-	✓	-	-	-	✓	✓	-	✓	-	1	-	-	1	✓	✓	-	-	-
TAD [6]	-	?	-	✓	-	✓	-	✓	✓	-	✓	-	any	-	-	1	✓	-	✓	-	✓
Liu *et al.* [7,8]	✓	-	-	✓	-	-	-	✓	✓	-	-	-	any	-	-	-	-	-	-	-	-
Shaknovich *et al.* [9]	?	?	-	✓	-	-	-	✓	✓	-	✓	-	?	-	-	-	-	-	-	-	-
Vrolijk *et al.* [10]	-	-	-	✓	-	-	-	✓	✓	✓	-	✓	any	-	-	-	-	-	-	-	-
CanSto [11]	✓	✓	-	✓	-	-	-	✓	✓	-	✓	-	2	-	-	1	✓	✓	-	-	-
TMAJ [12]	✓	✓	-	✓	-	✓	-	✓	✓	-	✓	-	any	-	-	2	✓	✓	-	✓	✓
Chen *et al.* [13,14]	?	?	-	✓	-	-	-	✓	✓	✓	?	✓	any	-	-	?	✓	?	?	✓	-
TmaDB [15]	✓	✓	-	✓	-	✓	-	✓	✓	-	✓	-	5	✓	-	1	✓	-	✓	✓	✓
Profiler [16]	✓	✓	-	✓	-	✓	-	✓	✓	-	✓	-	any	✓	-	?	✓	✓	✓	✓	-
TMABoost [17,18]	✓	✓	-	✓	-	✓	-	✓	✓	✓	✓	✓	any	✓	-	7	✓	-	✓	-	-
TissueChips [19]	-	✓	-	✓	-	-	-	✓	✓	✓	✓	✓	1	-	-	-	✓	-	-	-	-
VTM [20]	✓	✓	-	✓	-	✓	-	✓	✓	-	✓	-	?	-	-	2	✓	✓	✓	-	✓
Cruella [21]	✓	✓	-	✓	✓	-	-	✓	-	-	-	-	any	✓	-	1	✓	✓	✓	✓	-
Xperanto-TMA [22]	✓	✓	-	✓	-	-	-	✓	-	-	✓	-	43	✓	✓	>1	✓	✓	✓	✓	-
TAMEE [this study]	✓	✓	✓	✓	✓	✓	✓	✓	✓	✓	-	✓	any	✓	✓	>10	✓	✓	✓	✓	✓

1. Berman JJ, Edgerton ME, Friedman BA: **The tissue microarray data exchange specification: a community-based, open source tool for sharing tissue microarray data.** *BMC Med Inform Decis Mak* 2003, **3:**5.
2. Nohle DG, Ayers LW: **The tissue microarray data exchange specification: A document type definition to validate and enhance XML data.** *BMC Med Inform Decis Mak* 2005, **5:**12.
3. Deutsch EW, Ball CA, Bova GS, Brazma A, Bumgarner RE, Campbell D, Causton HC, Christiansen J, Davidson D, Eichner LJ et al.: **Development of the Minimum Information Specification for In Situ Hybridization and Immunohistochemistry Experiments (MISFISHIE).** *OMICS* 2006, **10:**205-208.
4. Bova GS, Parmigiani G, Epstein JI, Wheeler T, Mucci NR, Rubin MA: **Web-based tissue microarray image data analysis: initial validation testing through prostate cancer Gleason grading.** *Hum Pathol* 2001, **32:**417-427.
5. Manley S, Mucci NR, De Marzo AM, Rubin MA: **Relational database structure to manage high-density tissue microarray data and images for pathology studies focusing on clinical outcome: the prostate specialized program of research excellence model.** *Am J Pathol* 2001, **159:**837-843.
6. Coombes KR, Zhang L, Bueso-Ramos C, Brisbay S, Logothetis C, Roth J, Keating MJ, McDonnell TJ: **TAD: a web interface and database for tissue microarrays.** *Appl Bioinformatics* 2002, **1:**155-158.
7. Liu CL, Prapong W, Natkunam Y, Alizadeh A, Montgomery K, Gilks CB, van de RM: **Software tools for high-throughput analysis and archiving of immunohistochemistry staining data obtained with tissue microarrays.** *Am J Pathol* 2002, **161:**1557-1565.
8. Liu CL, Montgomery KD, Natkunam Y, West RB, Nielsen TO, Cheang MC, Turbin DA, Marinelli RJ, van de RM, Higgins JP: **TMA-Combiner, a simple software tool to permit analysis of replicate cores on tissue microarrays.** *Mod Pathol* 2005, **18:**1641-1648.
9. Shaknovich R, Celestine A, Yang L, Cattoretti G: **Novel relational database for tissue microarray analysis.** *Arch Pathol Lab Med* 2003, **127:**492-494.
10. Vrolijk H, Sloos W, Mesker W, Franken P, Fodde R, Morreau H, Tanke H: **Automated acquisition of stained tissue microarrays for high-throughput evaluation of molecular targets.** *J Mol Diagn* 2003, **5:**160-167.
11. Henshall S: **Tissue microarrays.** *J Mammary Gland Biol Neoplasia* 2003, **8:**347-358.
12. De Marzo AM, Morgan JD, Iacobuzio-Donahue C, Razzaque B, Faith DA: **TMAJ: Open Source Software to Manage a Tissue Microarray Database [abstract].** *Arch Pathol Lab Med* 2004, **128:**1094
13. Chen W, Foran DJ, Reiss M: **Unsupervised imaging, registration and archiving of tissue microarrays.** *Proc AMIA Symp* 2002,136-139.
14. Chen W, Reiss M, Foran DJ: **A prototype for unsupervised analysis of tissue microarrays for cancer research and diagnostics.** *IEEE Trans Inf Technol Biomed* 2004, **8:**89-96.
15. Sharma-Oates A, Quirke P, Westhead DR: **TmaDB: a repository for tissue microarray data.** *BMC Bioinformatics* 2005, **6:**218.
16. Kim R, Demichelis F, Tang J, Riva A, Shen R, Gibbs DF, Mahavishno V, Chinnaiyan AM, Rubin MA: **Internet-based Profiler system as integrative framework to support translational research.** *BMC Bioinformatics* 2005, **6:**304.
17. Dell'Anna R, Demichelis F, Barbareschi M, Sboner A: **An automated procedure to properly handle digital images in large scale tissue microarray experiments.** *Comput Methods Programs Biomed* 2005, **79:**197-208.
18. Demichelis F, Sboner A, Barbareschi M, Dell'Anna R: **TMABoost: an integrated system for comprehensive management of tissue microarray data.** *IEEE Trans Inf Technol Biomed* 2006, **10:**19-27.
19. Rabinovich A, Krajewski S, Krajewska M, Shabaik A, Hewitt SM, Belongie S, Reed JC, Price JH: **Framework for parsing, visualizing and scoring tissue microarray images.** *IEEE Trans Inf Technol Biomed* 2006, **10:**209-219.
20. Conway CM, O'Shea D, O'Brien S, Lawler DK, Dodrill GD, O'Grady A, Barrett H, Gulmann C, O'Driscoll L, Gallagher WM et al.: **The development and validation of the Virtual Tissue Matrix, a software application that facilitates the review of tissue microarrays on line.** *BMC Bioinformatics* 2006, **7:**256.
21. Cowan JD, Rimm DL, Tuck DP: **Cruella: developing a scalable tissue microarray data management system.** *Arch Pathol Lab Med* 2006, **130:**817-822.
22. Lee HW, Park YR, Sim J, Park RW, Kim WH, Kim JH: **The tissue microarray object model: a data model for storage, analysis, and exchange of tissue microarray experimental data.** *Arch Pathol Lab Med* 2006, **130:**1004-1013.
