# Supplementary material for: TAMEE: data management and analysis for tissue microarrays
Source: BMC Bioinformatics. 2007 Mar 7;8:81. doi: 10.1186/1471-2105-8-81 (PMC1838435; doi:10.1186/1471-2105-8-81)
Supplement: Additional File 2 — Sample core evaluation algorithm. Zip file containing Java source code of a sample core image evaluation algorithm and a description of the plug-in framework as the basis for the development of additional algorithms. [file 1471-2105-8-81-S2.zip › tmaMDA_CoreIntensityAlgorithm/documentation/TMA Algorithm Framework.pdf]

## Plug-in framework for core image evaluation algorithms

To integrate algorithms to evaluate core images in a flexible and extendable way, a plug-in framework has been implemented. The framework consists of the following components: (i) a message driven bean to instantiate an algorithm class and start the analysis (ii) an abstract algorithm bean, which defines the methods to be implemented and provides convenience functions which can be utilized by the derived algorithms, (iii) a flexible database model to manage the available algorithms and to store the image evaluation results and (iv) a web interface to deploy, manage and invoke the algorithms.

*Algorithm instantiation:* Depending of the number of core images comprising a section and the size of the images, the analysis can be quite time consuming. Therefore the analysis is performed asynchronously in the context of a Message Driven Bean (MDB) using the Java Messaging Service (JMS). An `ObjectMessage` object containing the algorithm name and the section or core ID is sent to the JMS "Algorithm". This triggers the instantiation of `ExecuteAlgorithmMDBBeanImpl` object where in turn an object of the relevant algorithm implementation is created based on the class name of the algorithm to be invoked and its start() method is called.

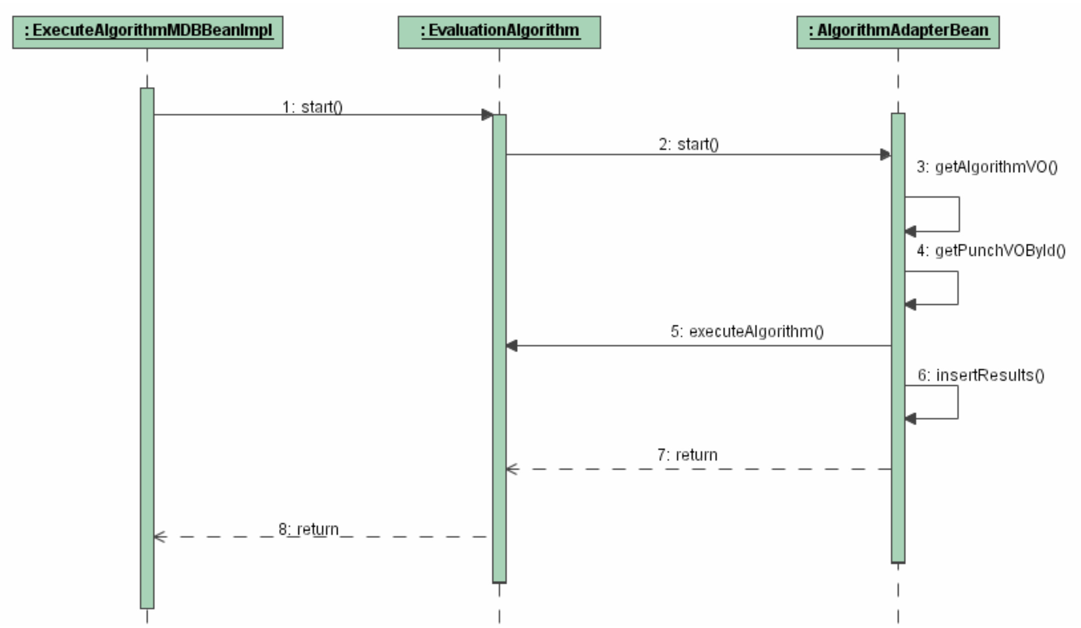


**Figure 1 – UML Sequence Diagram with the program flow during the evaluation of a single core image. The MDB instantiates the evaluation algorithm class and calls its start() method. In the start() method of the parent class the images related to the core are retrieved, the evaluation method is invoked and the results are stored in the database.**

*Algorithm adapter:* The abstract algorithm bean class `AlgorithmAdapterBean` is the basis for every newly implemented evaluation algorithm. New algorithms extend this class and have to implement the abstract method `executeAlgorithm()` defined in `AlgorithmAdapterBean` for the actual execution of the algorithm. This method is called for each core image belonging to a section. It receives an `AlgorithmAdapterData` object and a `HashTable` as parameters. The data object contains the images of a certain core together with their primary key and the hash table holds optional parameters for the execution of the algorithm. `executeAlgorithm()` returns a collection of `AlgorithmAdapterResult` objects, where each object contains the name of the result, the calculated result value and the primary key of the punch images the result is associated to.

Additionally the algorithm class has to overload the `start()` method, which is called when the analysis of a single core image or all cores of a section is invoked from the web interface or an applet. The `start()` method receives an `InputMessage` object as the parameter, which holds the section ID or core ID and the ID of the algorithm. It has to call the `start()` method of the parent, where the actual analysis is performed. The complete flow of operation is shown in Figure 1.
Aside the methods `start()`and `executeAlgorithm()` the algorithm class has to implement the methods `getVersion()` to retrieve the version number of the implementation of the algorithm and is`Startable()` to determine whether the algorithm can be started directly via the web interface. Currently only the algorithm for the combined evaluation of a multiplex IF experiment [1] has to be started from within an applet, all others are invoked via the web interface.

*Database model of the plug-in framework:* Results of the evaluation of a core image include "boolean" outcomes like "present" / "not present", scores with various levels (e.g. "0", "1", "2" "3" or FIXXME), values on a continuous scale or percentages. To accommodate the diverse types of possible results, a flexible database model has been designed based on the Entity-Attribute Value (EAV) paradigm [2]. Within the presented implementation, the "Entity" is a certain core together with its images which have been evaluated, the "Attribute" is the algorithm used for the evaluation of the core image(s) and the "Values" are the results of the analysis run (Figure 2). In contrast to the general EAV model, where the values can have different data types, a simplified version has been implemented with all values stored as strings regardless of their actual data type.

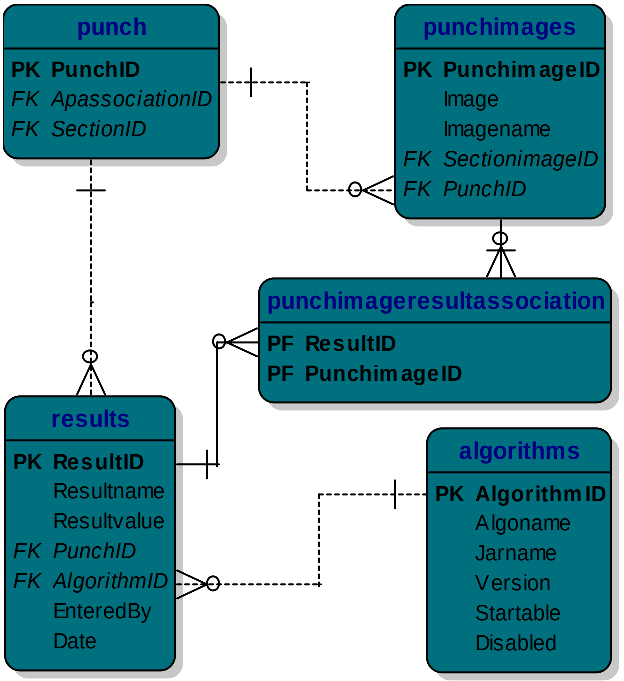


**Figure 2 – Database tables related to the flexible and extensible storage of evaluation results. The punch table defines available cores which are related to a certain section and a certain position. Associated images and results are stored in the punchimages and results tables respectively. Each result originates from a certain evaluation algorithm in the algorithms table.**

*Algorithm web interface:* The web interface to manage the evaluation algorithms consists of three parts. The first one allows the deployment of a new algorithm, which is immediately available for use after the upload (Figure 3a). Display and management of existing algorithms is provided by the algorithm report screen (Figure 3b). Algorithm invocation for a certain

section is provided from within the "Start Algorithm" screen (Figure 3c). Additionally the evaluation results can be queried and displayed (Figure 3d).

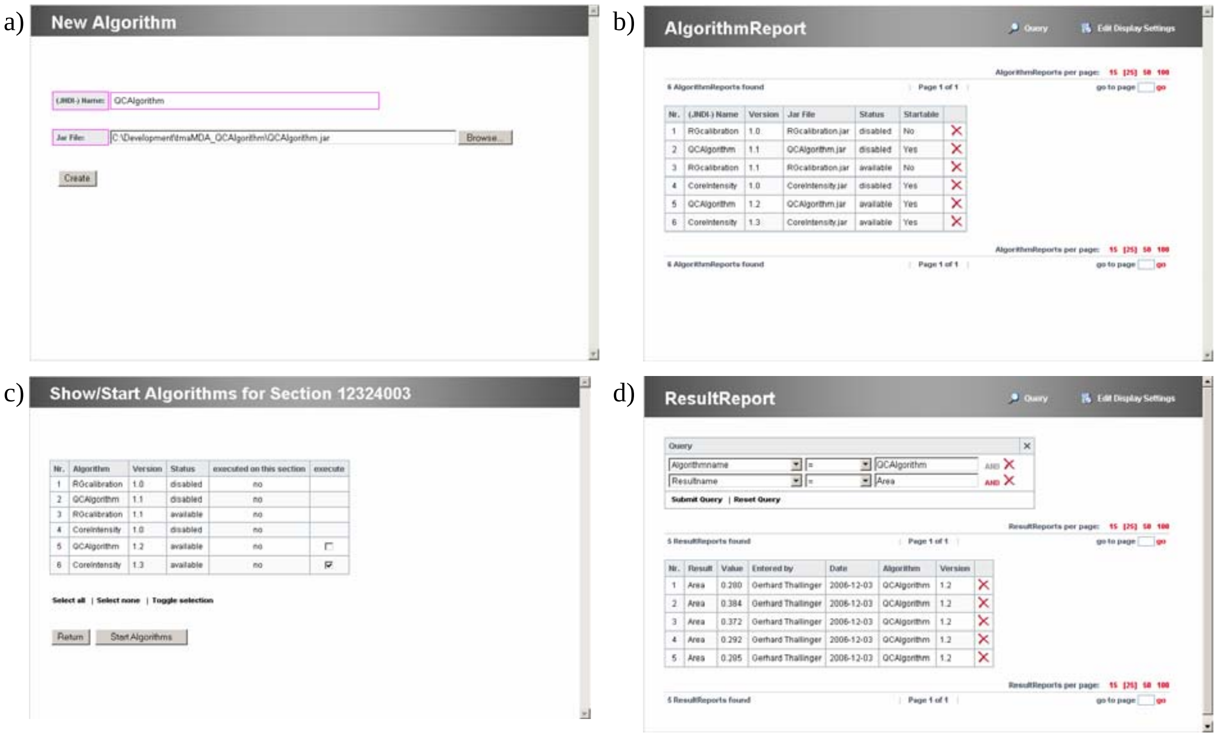


**Figure 3 – Web interface for (a) algorithm deployment, b) algorithm management, c) algorithm invocation and d) querying and display of results.**

# References


1. Pauritsch EM: **Mathematische Modellbildung bei mehrfachgefärbten Gewebeproben in der Immunhistochemie.** *Master's Thesis.* Graz University of Technology, Department of Mathematics C; 2003.
2. Johnson SB: **Generic data modeling for clinical repositories.** *J Am Med Inform Assoc* 1996, **3:**328-339.
