# Supplementary figures and images for: TAMEE: data management and analysis for tissue microarrays
Source: BMC Bioinformatics. 2007 Mar 7;8:81. doi: 10.1186/1471-2105-8-81 (PMC1838435; doi:10.1186/1471-2105-8-81)

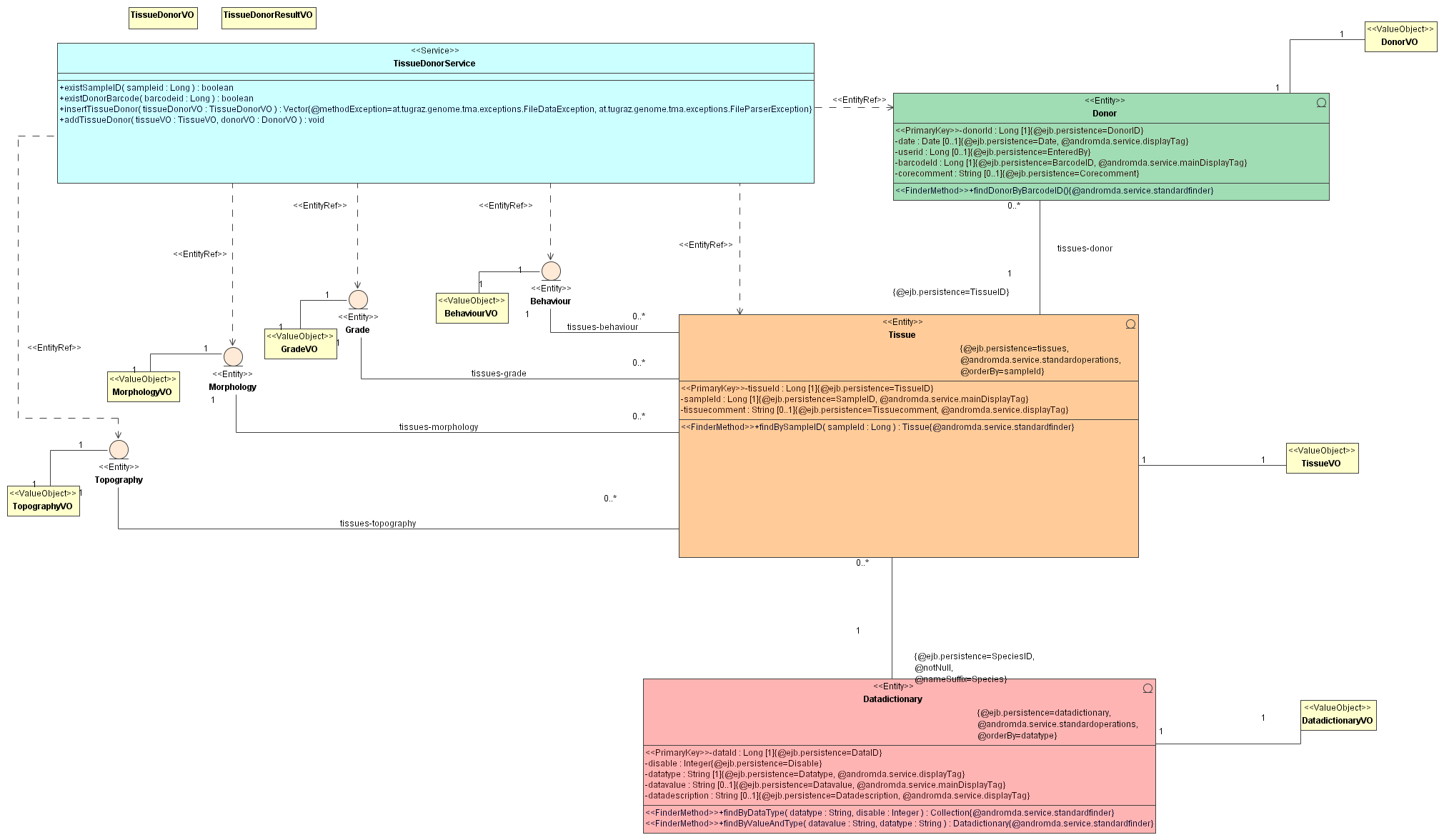

Supplement: Additional File 3 — TAMEE UML model. Zip file containing the TAMEE UML model comprising the entity and the service diagrams. [file 1471-2105-8-81-S3.zip › Class_Diagram__TissueDonorService.png]

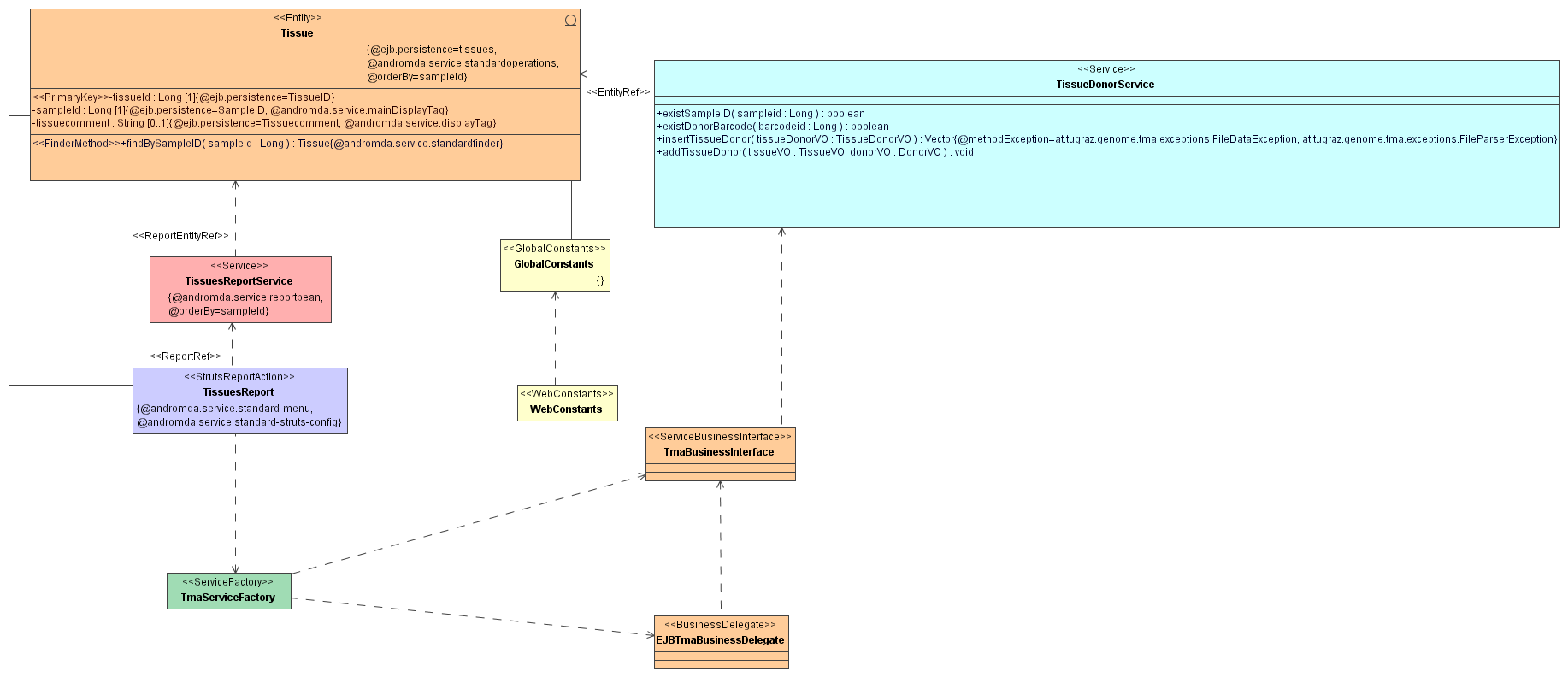

Supplement: Additional File 3 — TAMEE UML model. Zip file containing the TAMEE UML model comprising the entity and the service diagrams. [file 1471-2105-8-81-S3.zip › Class_Diagram__TissuesReport.png]

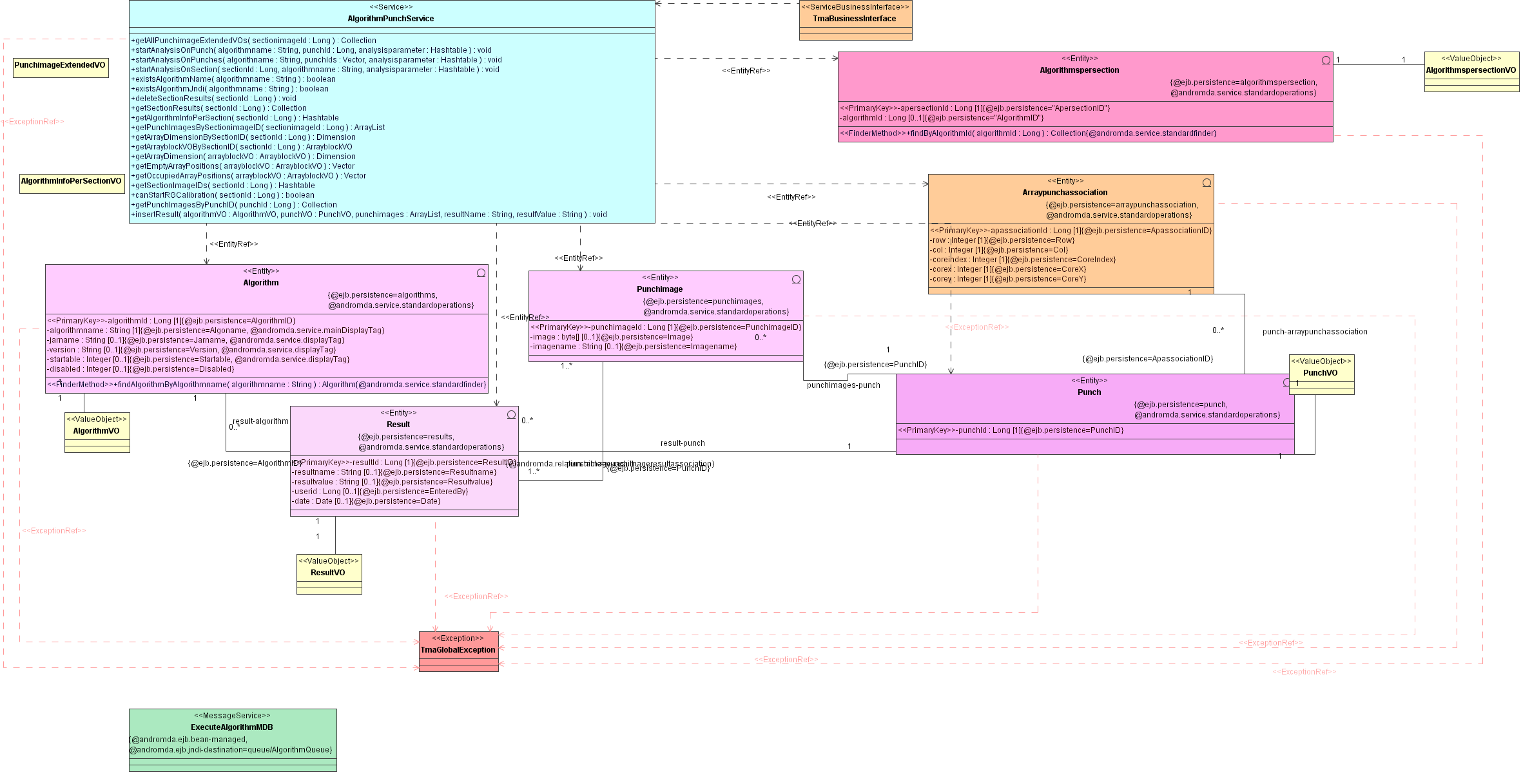

Supplement: Additional File 3 — TAMEE UML model. Zip file containing the TAMEE UML model comprising the entity and the service diagrams. [file 1471-2105-8-81-S3.zip › Class_Diagram__AlgorithmPunchService.png]

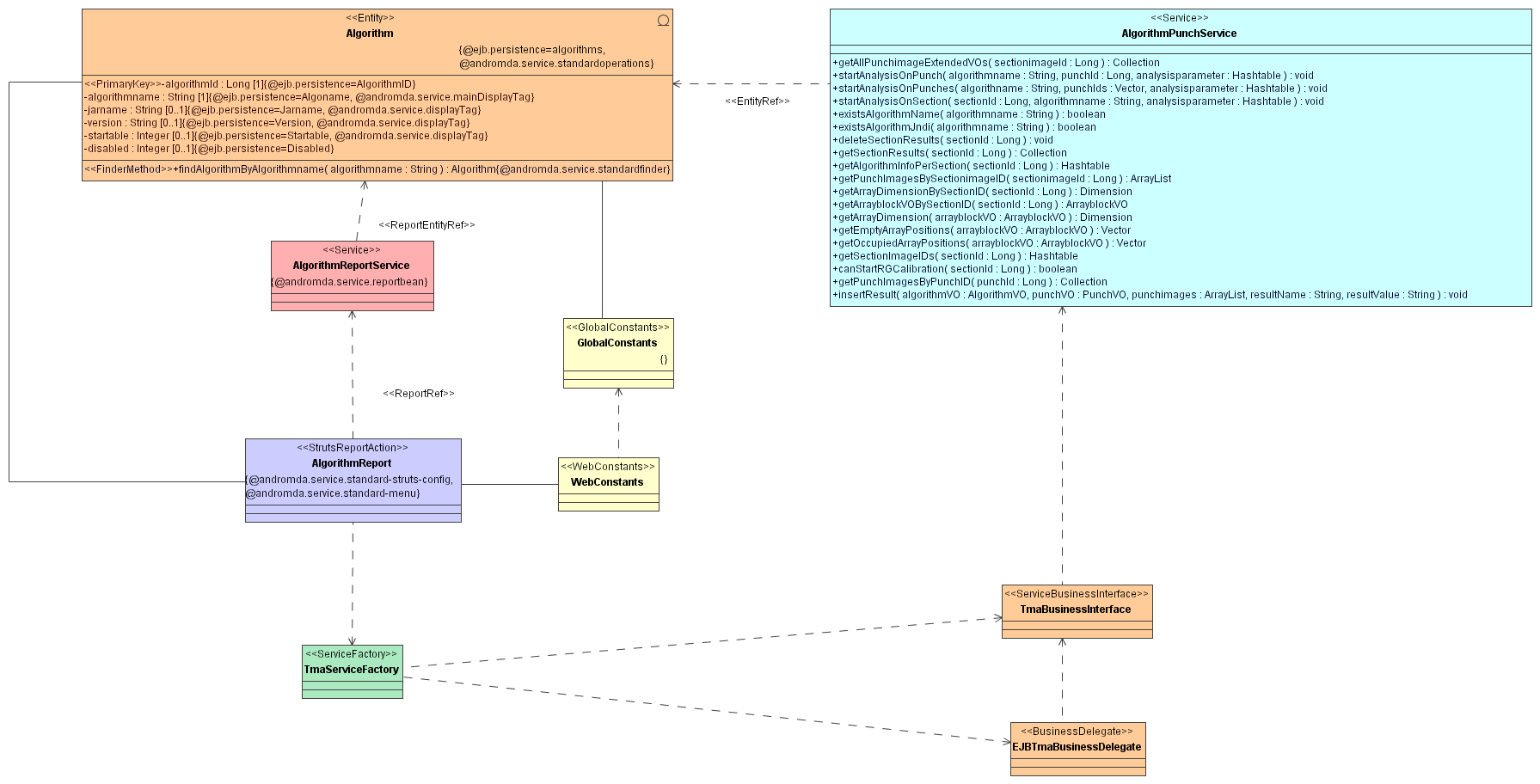

Supplement: Additional File 3 — TAMEE UML model. Zip file containing the TAMEE UML model comprising the entity and the service diagrams. [file 1471-2105-8-81-S3.zip › Class_Diagram__AlgorithmReport.png]

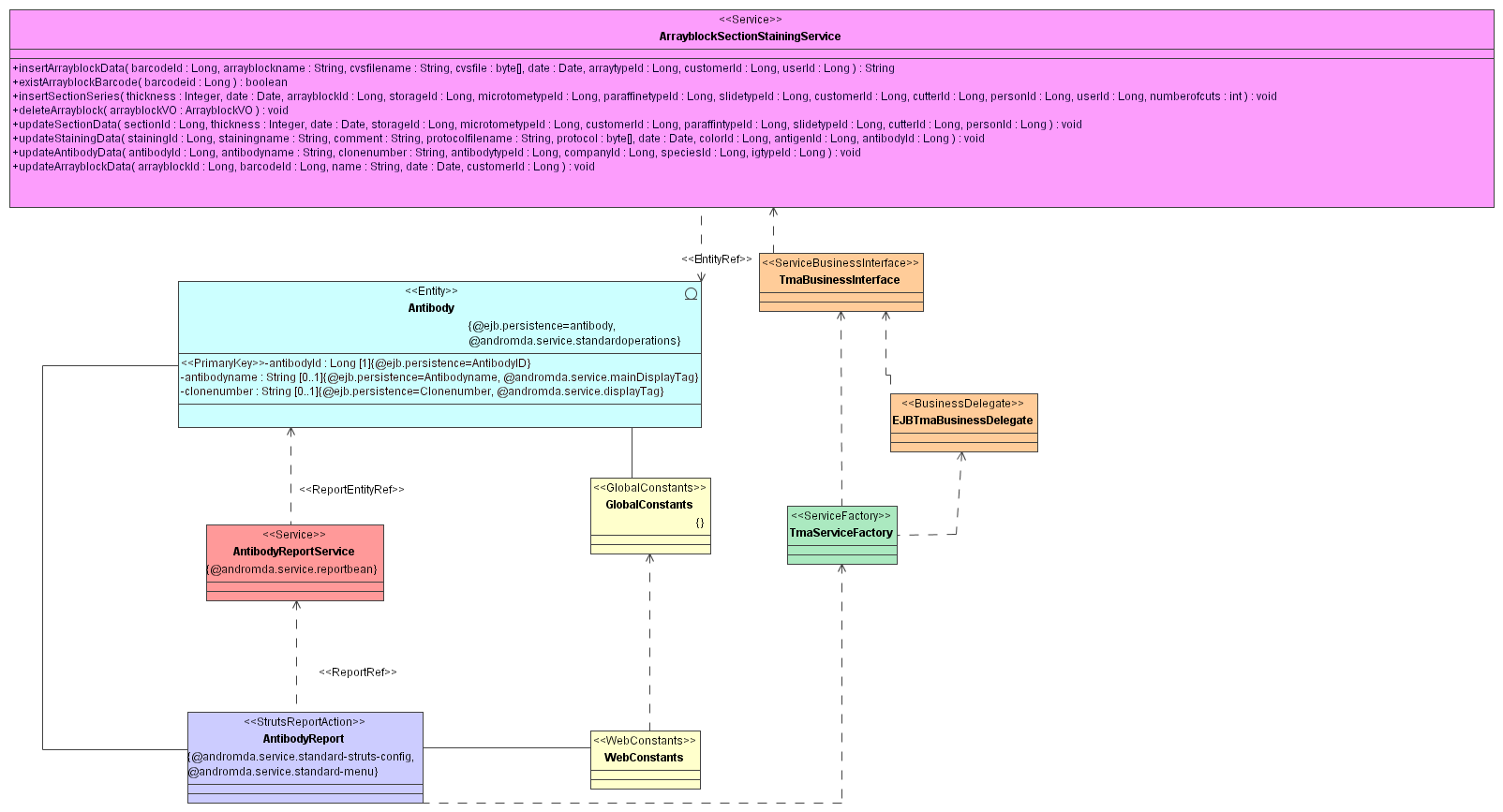

Supplement: Additional File 3 — TAMEE UML model. Zip file containing the TAMEE UML model comprising the entity and the service diagrams. [file 1471-2105-8-81-S3.zip › Class_Diagram__AntibodyReport.png]

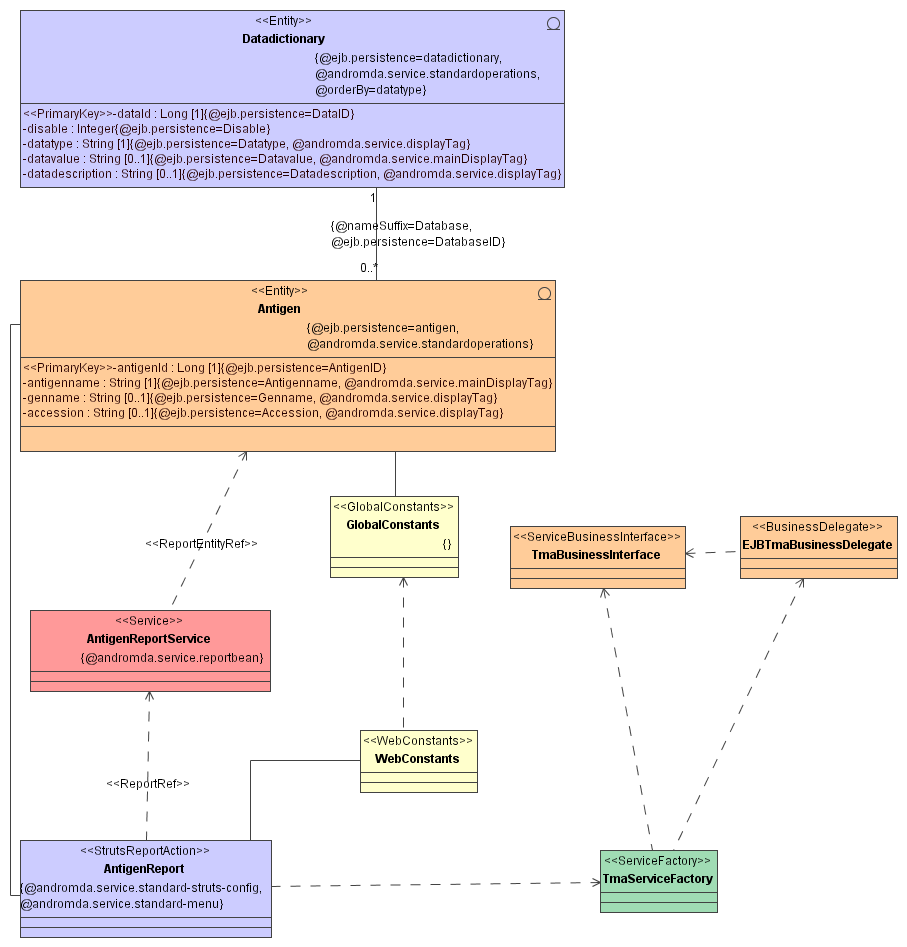

Supplement: Additional File 3 — TAMEE UML model. Zip file containing the TAMEE UML model comprising the entity and the service diagrams. [file 1471-2105-8-81-S3.zip › Class_Diagram__AntigenReport.png]

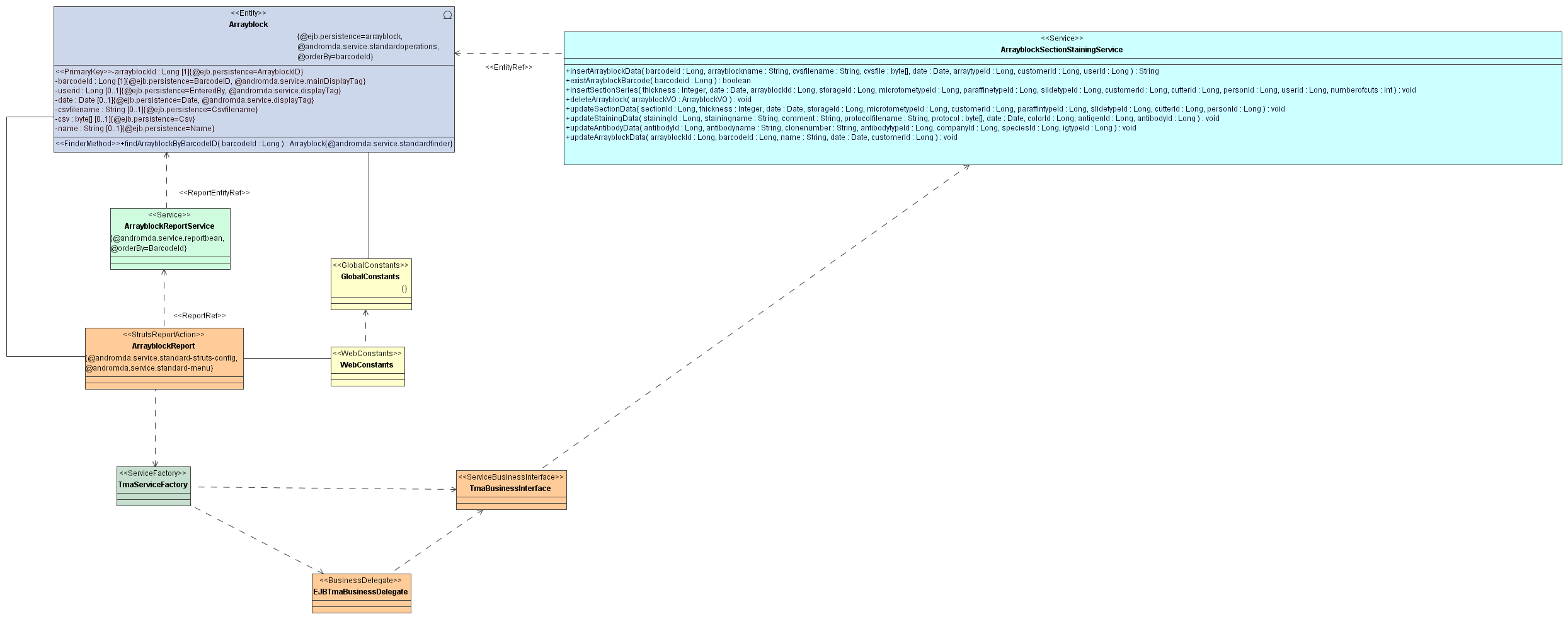

Supplement: Additional File 3 — TAMEE UML model. Zip file containing the TAMEE UML model comprising the entity and the service diagrams. [file 1471-2105-8-81-S3.zip › Class_Diagram__ArrayblockReport.png]

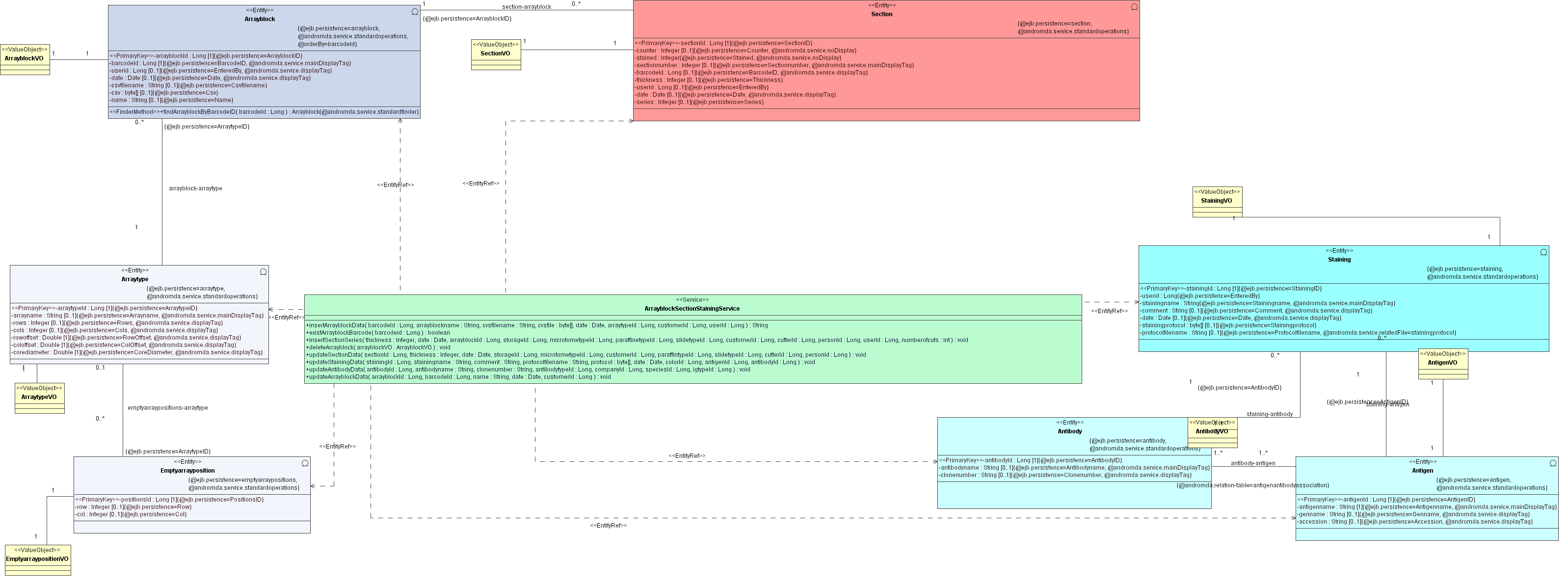

Supplement: Additional File 3 — TAMEE UML model. Zip file containing the TAMEE UML model comprising the entity and the service diagrams. [file 1471-2105-8-81-S3.zip › Class_Diagram__ArrayblockSectionStainigService.png]

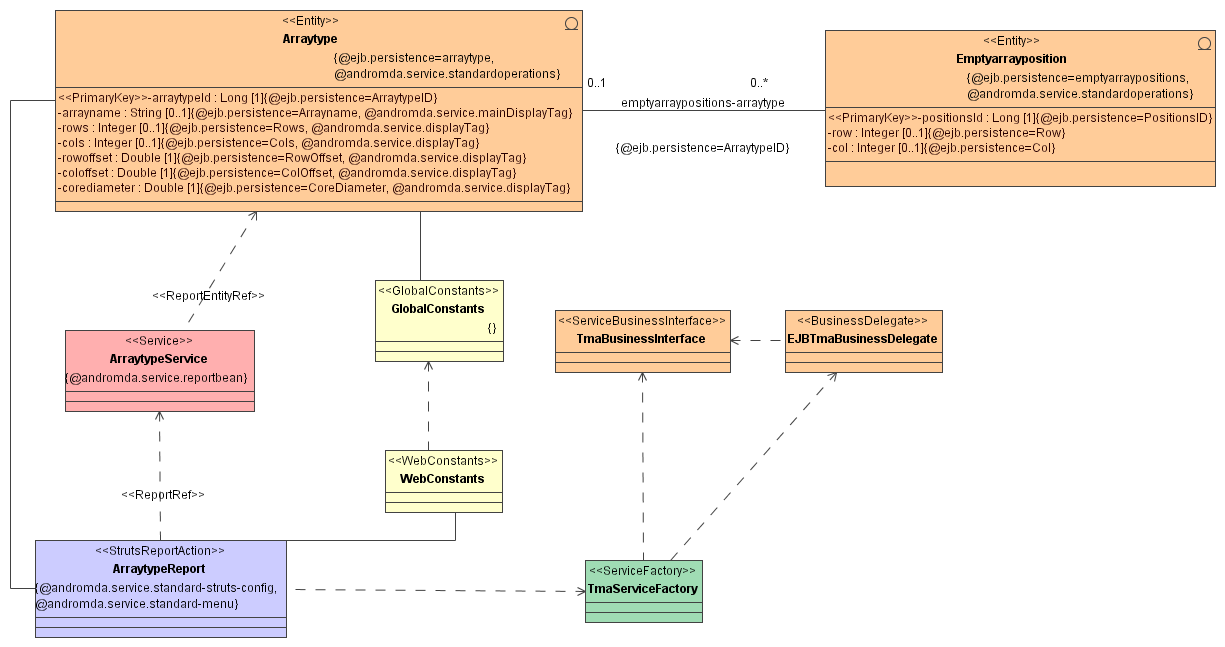

Supplement: Additional File 3 — TAMEE UML model. Zip file containing the TAMEE UML model comprising the entity and the service diagrams. [file 1471-2105-8-81-S3.zip › Class_Diagram__ArraytypeReport.png]

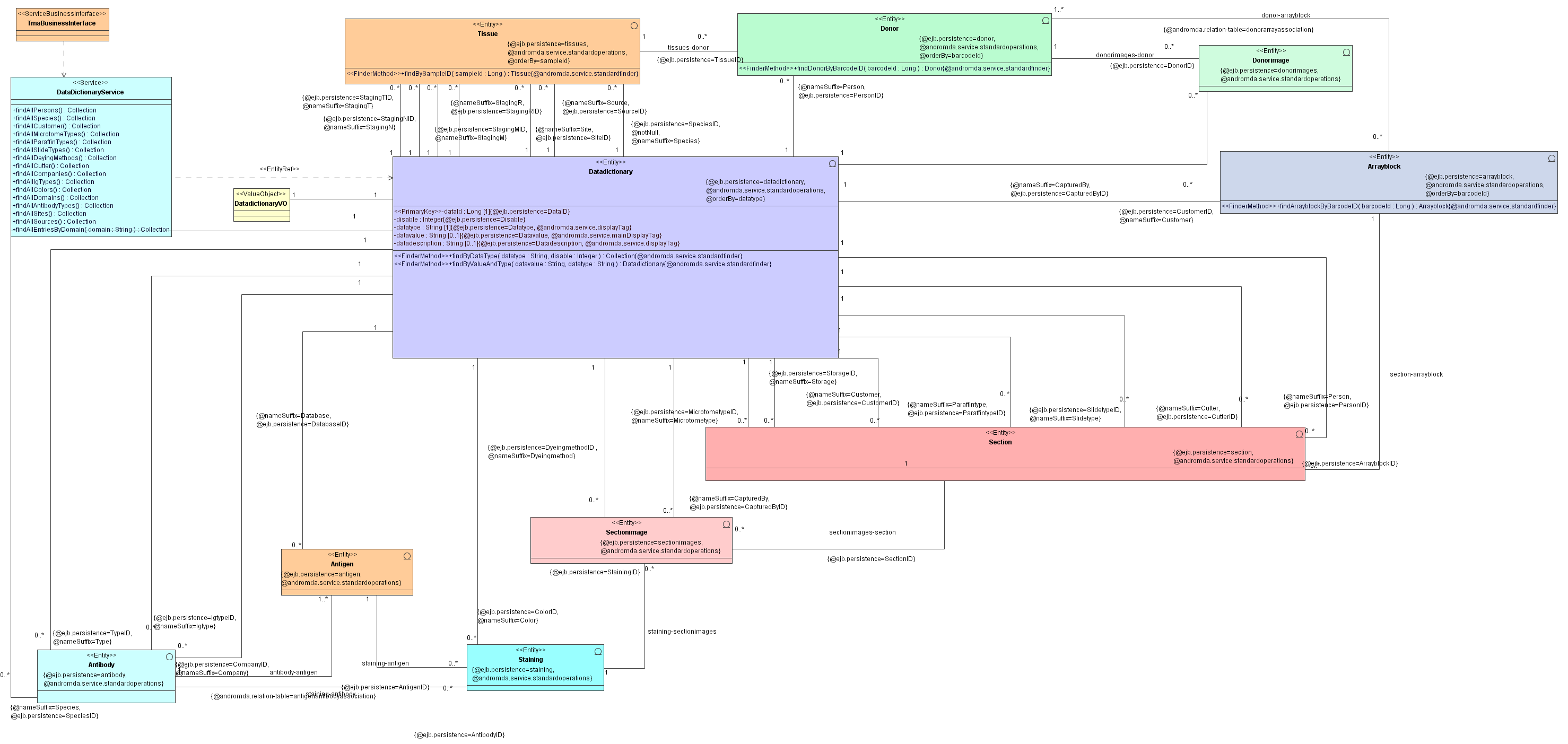

Supplement: Additional File 3 — TAMEE UML model. Zip file containing the TAMEE UML model comprising the entity and the service diagrams. [file 1471-2105-8-81-S3.zip › Class_Diagram__DataDictionary.png]

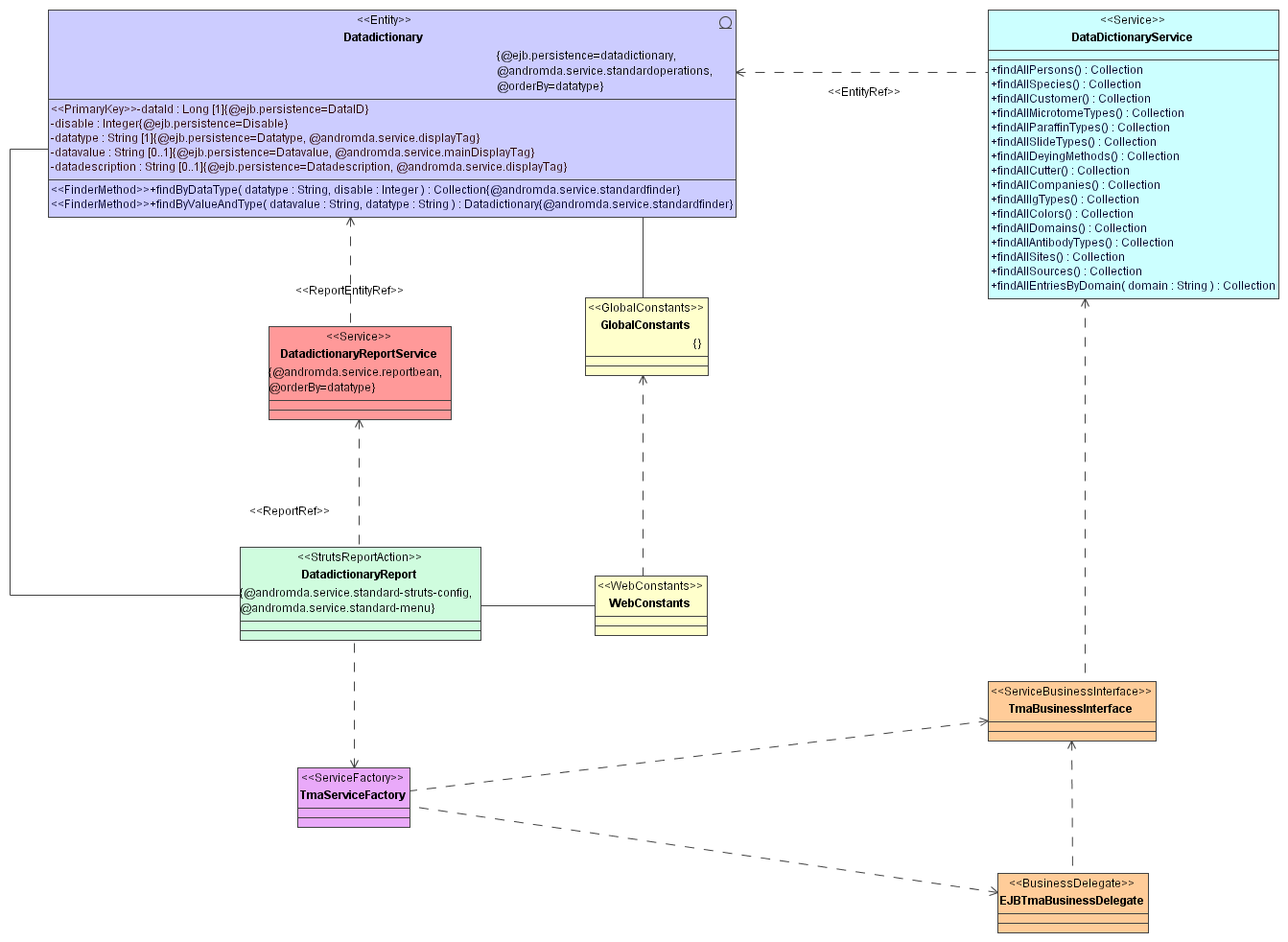

Supplement: Additional File 3 — TAMEE UML model. Zip file containing the TAMEE UML model comprising the entity and the service diagrams. [file 1471-2105-8-81-S3.zip › Class_Diagram__DatadictionaryReport.png]

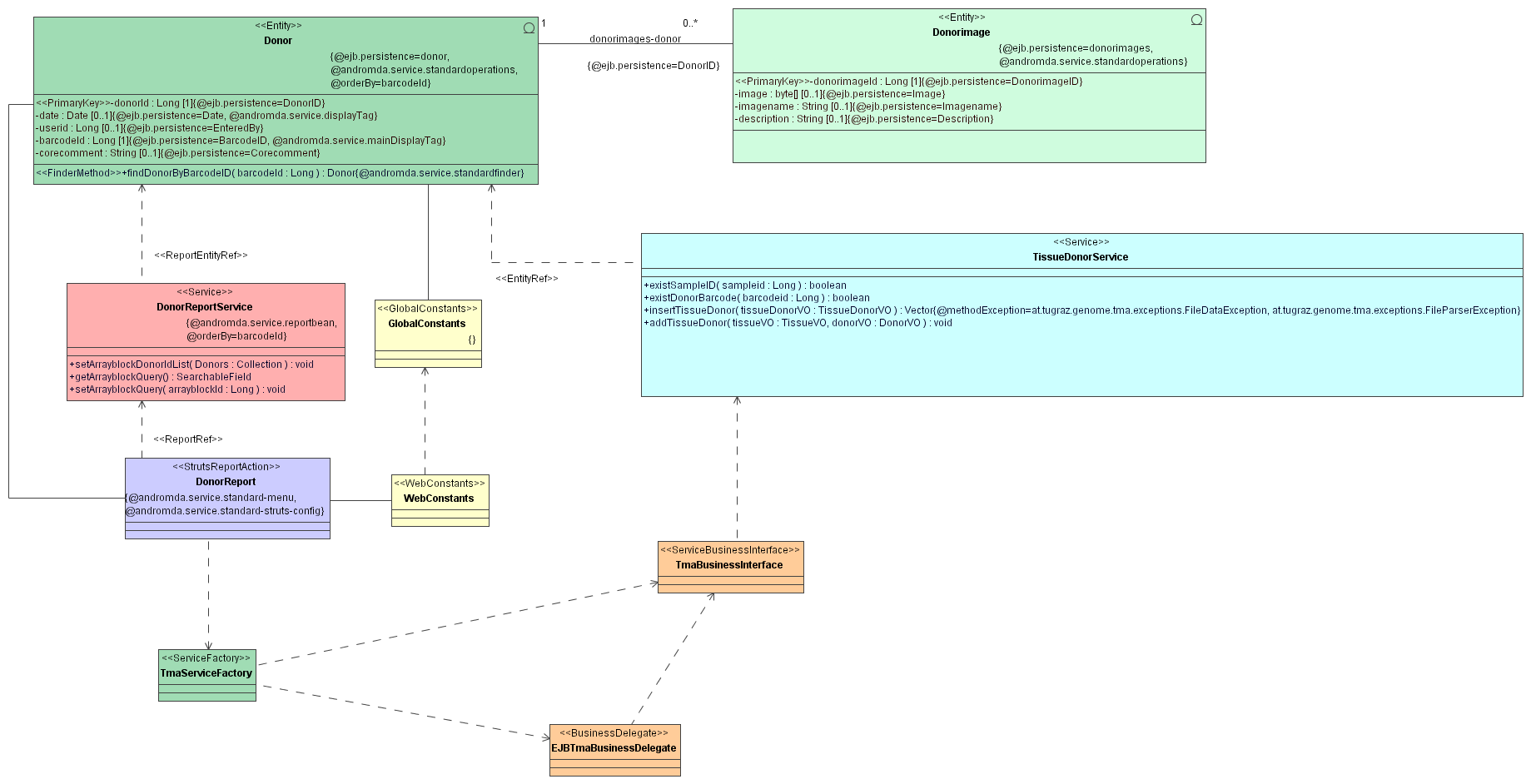

Supplement: Additional File 3 — TAMEE UML model. Zip file containing the TAMEE UML model comprising the entity and the service diagrams. [file 1471-2105-8-81-S3.zip › Class_Diagram__DonorReport.png]

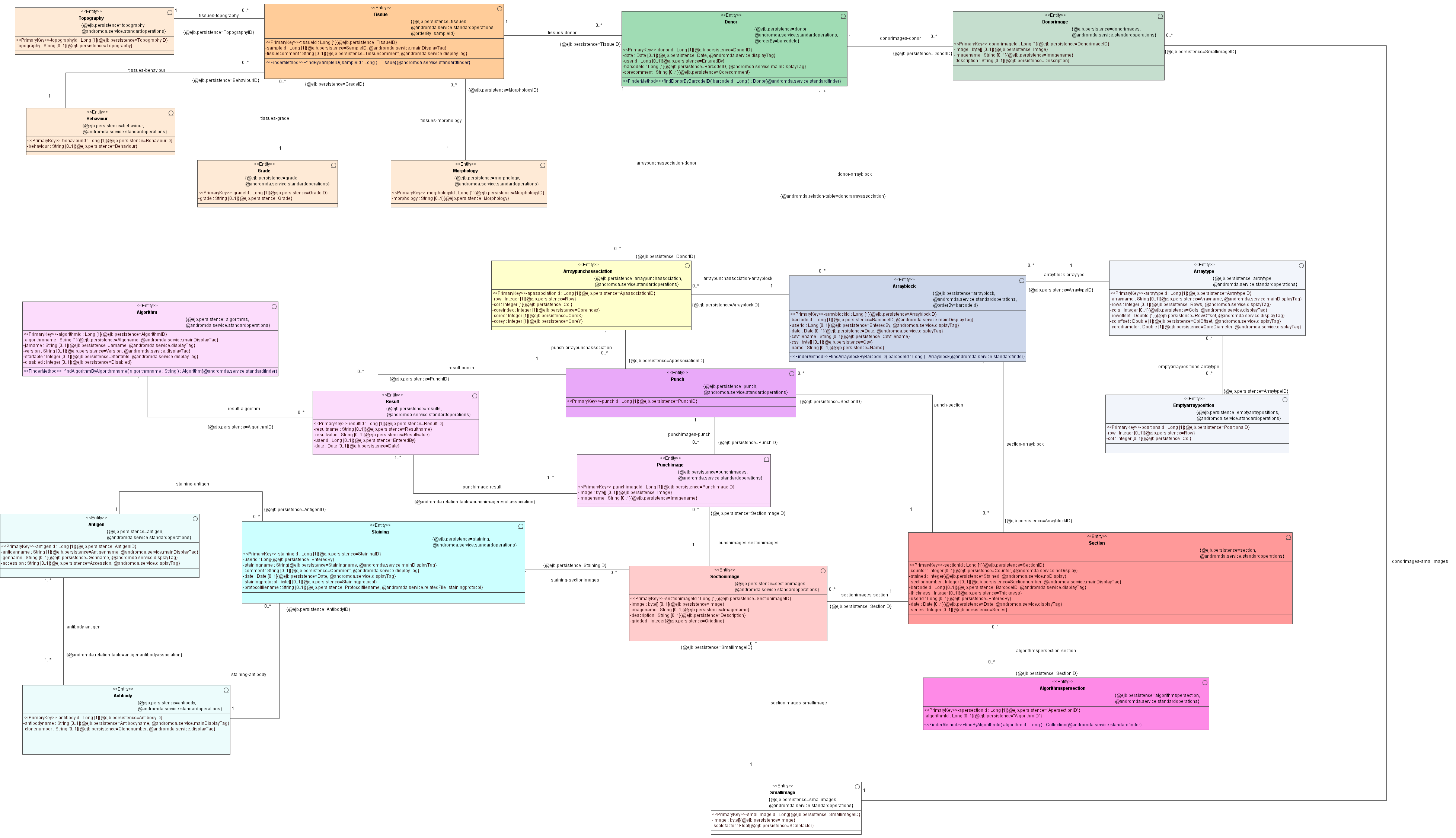

Supplement: Additional File 3 — TAMEE UML model. Zip file containing the TAMEE UML model comprising the entity and the service diagrams. [file 1471-2105-8-81-S3.zip › Class_Diagram__Entity.png]

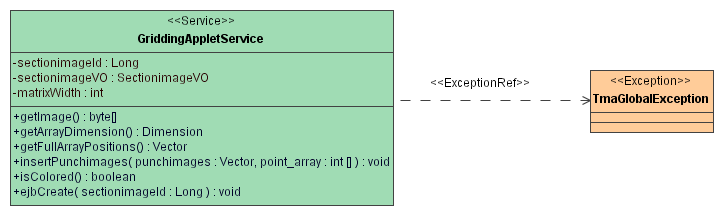

Supplement: Additional File 3 — TAMEE UML model. Zip file containing the TAMEE UML model comprising the entity and the service diagrams. [file 1471-2105-8-81-S3.zip › Class_Diagram__GriddingAppletService.png]

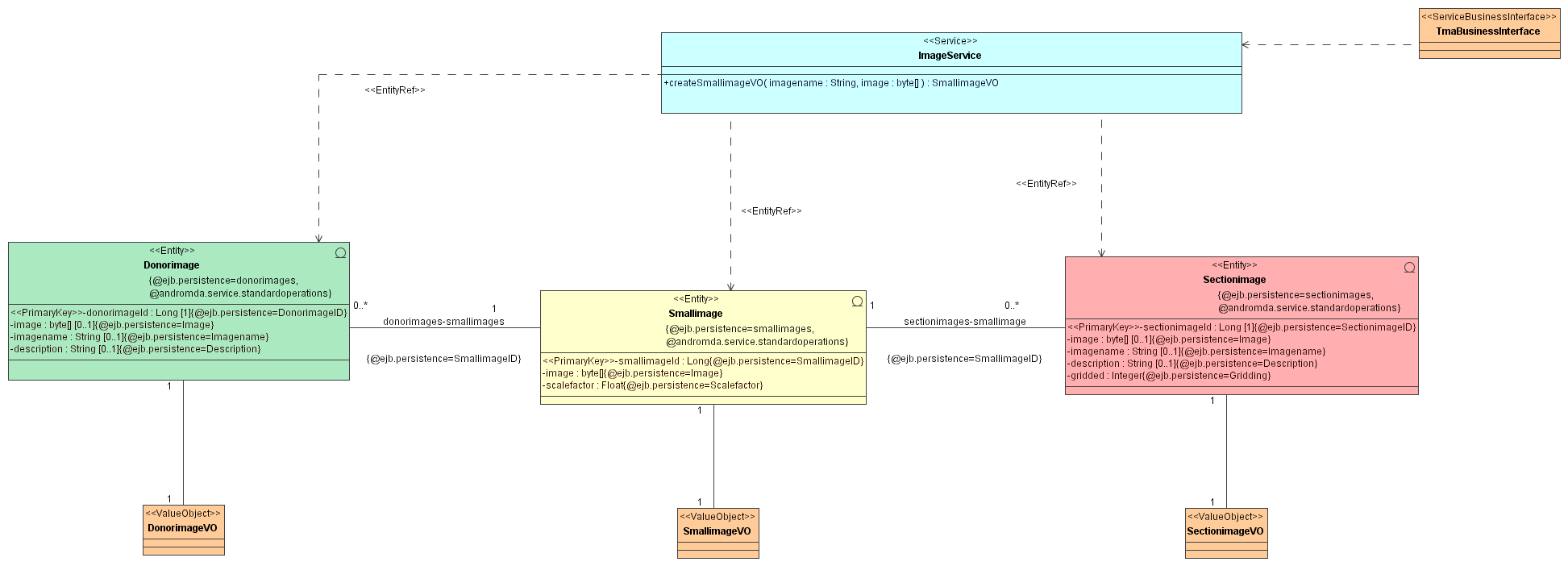

Supplement: Additional File 3 — TAMEE UML model. Zip file containing the TAMEE UML model comprising the entity and the service diagrams. [file 1471-2105-8-81-S3.zip › Class_Diagram__ImageService.png]

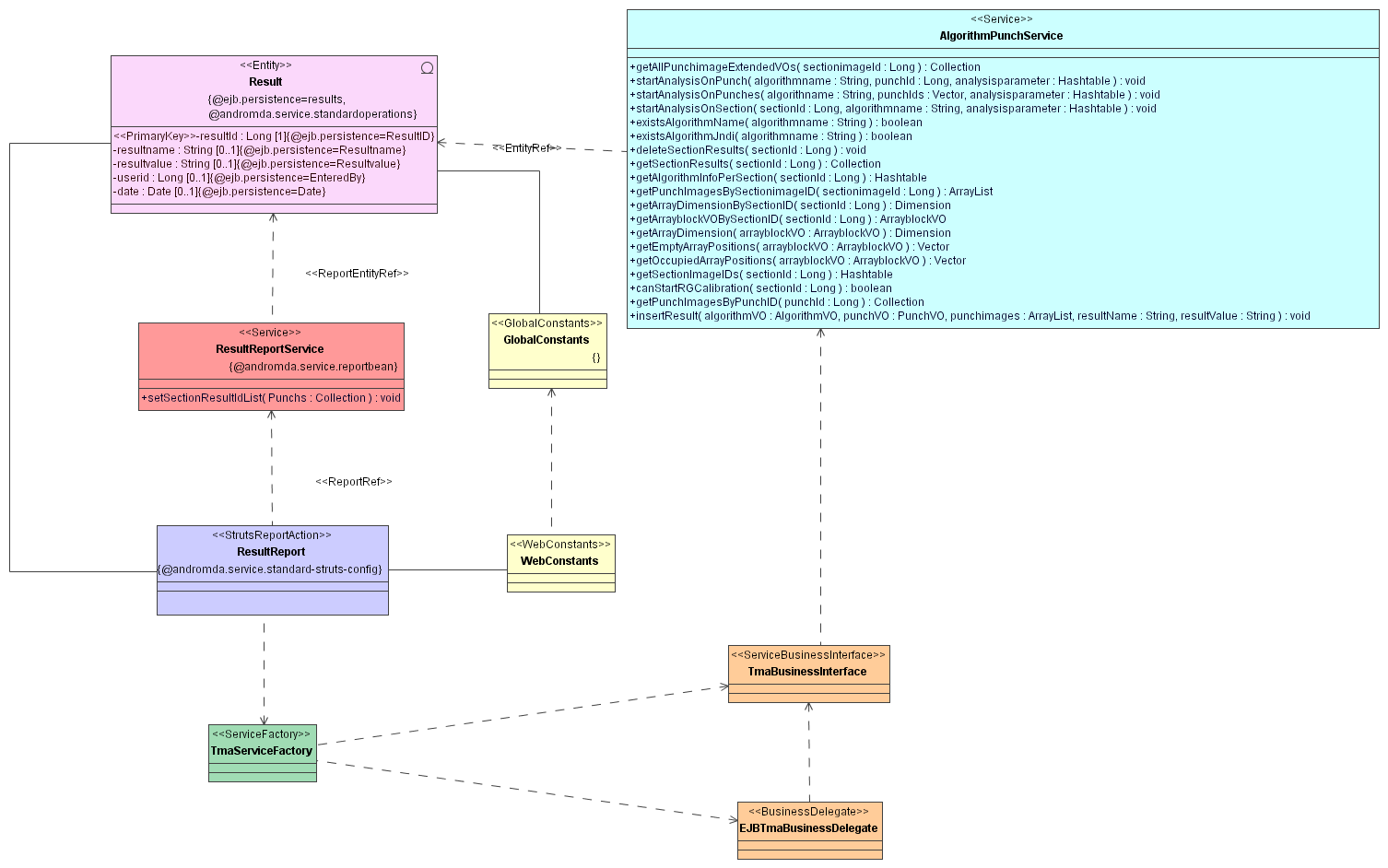

Supplement: Additional File 3 — TAMEE UML model. Zip file containing the TAMEE UML model comprising the entity and the service diagrams. [file 1471-2105-8-81-S3.zip › Class_Diagram__ResultReport.png]

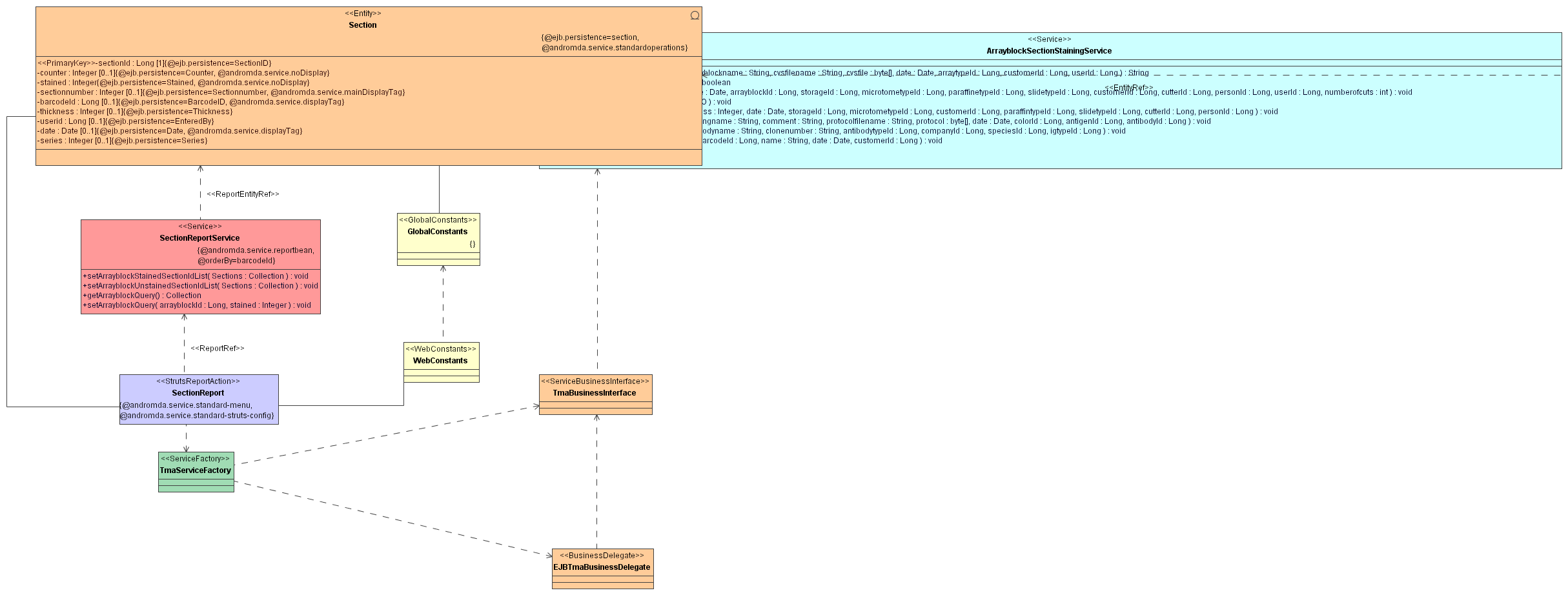

Supplement: Additional File 3 — TAMEE UML model. Zip file containing the TAMEE UML model comprising the entity and the service diagrams. [file 1471-2105-8-81-S3.zip › Class_Diagram__SectionReport.png]

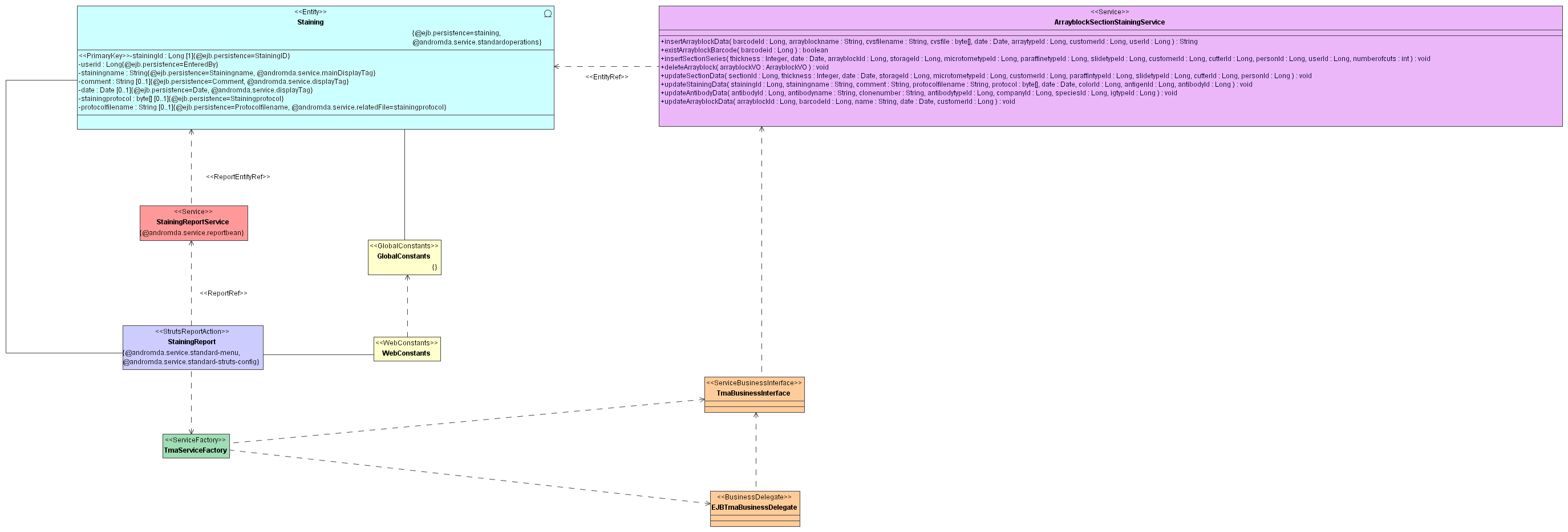

Supplement: Additional File 3 — TAMEE UML model. Zip file containing the TAMEE UML model comprising the entity and the service diagrams. [file 1471-2105-8-81-S3.zip › Class_Diagram__StainingReport.png]

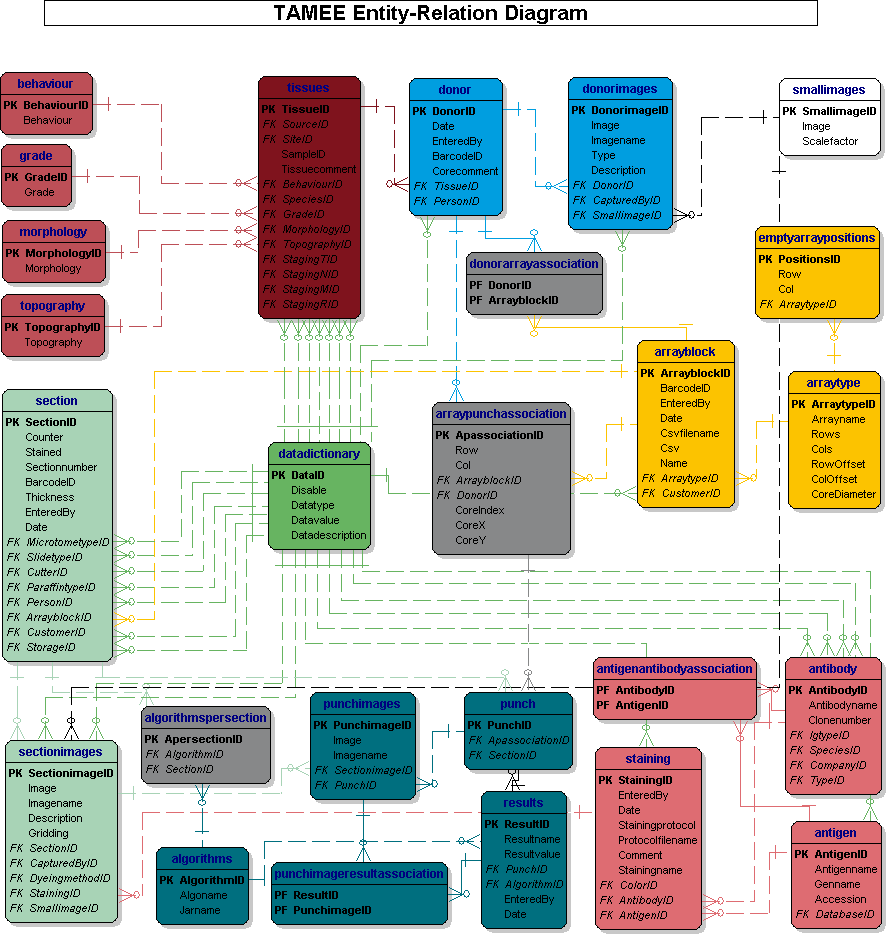

Supplement: Additional File 4 — TAMEE entity-relation diagram. Bitmap file containing the TAMEE entity relation diagram. [file 1471-2105-8-81-S4.bmp]
